# 4,5-Dicyano-1,2,3-Triazole—A Promising Precursor for a New Family of Energetic Compounds and Its Nitrogen-Rich Derivatives: Synthesis and Crystal Structures

**DOI:** 10.3390/molecules26216735

**Published:** 2021-11-07

**Authors:** Wenli Cao, Jian Qin, Jianguo Zhang, Valery P. Sinditskii

**Affiliations:** 1 State Key Laboratory of Explosion Science and Technology, Beijing Institute of Technology, Beijing 100081, China; li200806822@163.com (W.C.); qinjian0904@163.com (J.Q.); 2 China North Chemical Research Academy Group Co., LTD., Beijing 100089, China; 3 Chemical Engineering Department, Mendeleev University of Chemical Technology, 9 Miusskaya Sq., 125047 Moscow, Russia; vps@muctr.ru

**Keywords:** 1,2,3-triazole, 4,5-dicyano-1,2,3-triazole, Hofmann rearrangement, X-ray single crystal diffraction, high-nitrogen content

## Abstract

The nitrogen-rich compounds and intermediates with structure of monocyclic, bicyclic, and fused rings based on 1,2,3-triazole were synthesized and prepared by using a promising precursor named 4,5-dicyano-1,2,3-triazole, which was obtained by the cyclization reaction of diaminomaleonitrile. Their structure and configurational integrity were assessed by Fourier transform-infrared spectroscopy (FT-IR), mass spectrometry (MS), and elemental analysis (EA). Additionally, fourteen compounds were further confirmed by X-ray single crystal diffraction. Meanwhile, the physical properties of four selected compounds (**3**·H_2_O, **6**·H_2_O, **10**·H_2_O, and **16**) including thermal stability, detonation parameters, and sensitivity were also estimated. All these compounds could be considered to construct more abundant 1,2,3-triazole-based neutral energetic molecules, salts, and complex compounds, which need to continue study in the future in the field of energetic materials.

## 1. Introduction

Triazoles (1,2,3-triazole and 1,2,4-triazole) are electron-rich aromatic heterocycles. They also accept protons and metal ions easily, so supramolecular systems can be formed by coordinate bond, hydrogen bond, ion-dipole interaction, π-π stacking interaction, hydrophobic interaction, and Van der Waals forces. Accordingly, triazoles have extensive applications in medicinal chemistry [1], biological sciences [2], polymer material [3], and agriculture [4]. For instance, a series of novel conjugated porous polymers represented by 1,4-bis(3,5-di(thiophen-2-yl)phenyl)-1*H*-1,2,3-triazole were designed and synthesized, and the results revealed that these polymers taking 1,2,3-triazole as the acceptor unit exhibited prominent photocatalytic activities toward organic chemical transformations [5]. As the synthesis of 1,2,3-triazole can be easily and efficiently realized via click chemistry, 1,2,3-triazole-based derivatives have aroused growing attention for plenty of studies in recent years.

Most of the 1,2,3-triazoles have been known in the fields mentioned above. However, only few studies of 1,2,3-triazoles derivatives are reported in the field of energetic materials. At present, the research of energetic materials are concentrate on nitrogen-rich heterocyclic compounds which are furazan [6,7,8], 1,2,4-triazole [9,10,11], and tetrazole [12,13,14]. However, 1,2,3-triazole is a surprisingly stable structure compared to other organic compounds with three contiguous nitrogen atoms [15]. Notably, the 1,2,3-triazole are reported to be more thermally stable and higher heat of formation (240 kJ mol^−1^) than 1,2,4-triazole (182 kJ mol^−1^), owing to the special presence of a N-N-N (N3) structure [16,17,18]. Consequently, the detonation performance and thermal stability of energetic compounds could be enhanced simultaneously by combining 1,2,3-triazole with other heterocycles into one single energetic molecule. Such as, 4,5-bis(4,5-diamine-1,2,4-triazole-3-yl)-2*H*-1,2,3-triazole was designed and a series of energetic salts were synthesized by Gu et al. [19] The results indicated that it has the potential application in gas generating composition. Subsequently, the nitration product 4,5-bis(5-nitramino-1,2,4-triazole-3-yl)-2*H*-1,2,3-triazole and oxidized product 4,5-bis(5-nitro-1*H*-1,2,4-triazol-3-yl)-2*H*-1,2,3-triazole were also prepared and discovered the same potential that the carbohydrazide salt and triaminoguanidine salt of 4,5-bis(5-nitramino-1,2,4-triazole-3-yl)-2*H*-1,2,3-triazole could be useful as green gas generant ingredients [20]. In addition, the 1,2,3-triazole motif was also be used to obtain the high-performance energetic materials. A typical example, (5-nitro-2*H*-1,2,3-triazol-4-yl)-1*H*-tetrazol-5-amine (d = 1.86 g·cm^−3^, P = 33.71 GPa, D = 9153 m·s^−1^, T_d_ = 308 °C, IS = 40 J, FS = 360 N) and its hydroxylammonium salt (d = 1.80 g·cm^−3^, P = 34.75 GPa, D = 9482 m·s^−1^, T_d_ = 242 °C, IS = 40 J, FS = 360 N) exhibited excellent detonation properties [21]. Another example, two regioisomeric energetic molecules 1,2-bis(4-nitro-1*H*-1,2,3-triazol-1-yl)diazene and 1,2-bis(4-nitro-1*H*-1,2,3triazol-2-yl)diazene, which contain a linear nitrogen-atom chain and a branched nitrogen-atom chain, respectively, showed comparable detonation parameters, with a detonation velocity (8916–8917 m·s^−1^) and detonation pressure (33.49–33.53 GPa), respectively [22]. Apart from the above-mentioned compounds, other related work including preparation, properties, and applications based on 1,2,3-triazole derivatives were also explored and reported recently [23,24,25]. Therefore, the synthesis, crystal structures confirmation, and characterization of nitrogen-rich 4,5-substituted 1,2,3-triazole-based derivatives has important research significance.

In the present work, diaminomaleonitrile was used as the starting material to prepare 4,5-dicyano-1,2,3-triazole. This compound acted as a precursor, from which a proposed series of nitrogen-rich 1,2,3-triazole-based derivatives was synthesized. All these compounds were fully characterized by Fourier transform-infrared spectroscopy (FT-IR), mass spectrometry (MS), and elemental analysis (EA). Additionally, many of them were further confirmed by single-crystal X-ray diffraction. The thermal decomposition behavior was further investigated using differential scanning calorimetry (DSC) under nitrogen atmosphere. The energetic properties and mechanical sensitivity were also evaluated to better understand the structure–property relationships.

## 2. Results and Discussion

### 2.1. Synthetic Procedures and Structures

**Scheme 1:**
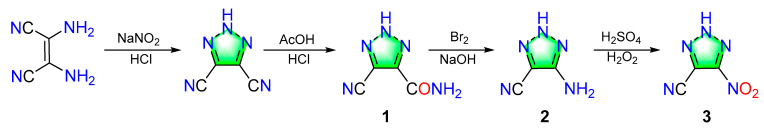
Synthesis of compounds **1**–**3**.

**Figure 1:**
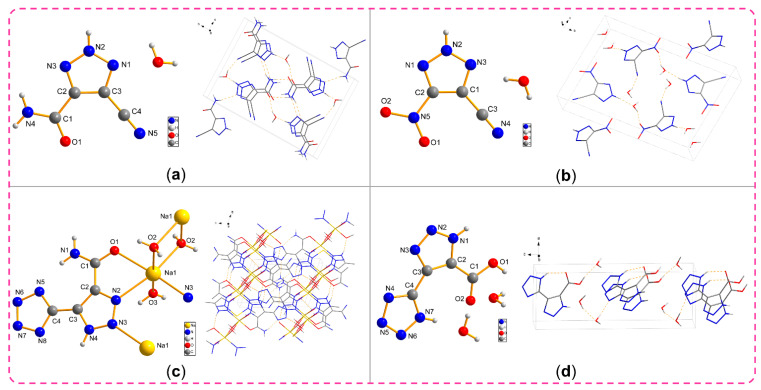
The crystal structure and crystal packing of compounds **1**·H_2_O (**a**), **3**·H_2_O (**b**), sodium salt **S4** (**c**), and **6**·2H_2_O (**d**).

There was a possible route to synthesize the new nitrogen-rich compound 4-cyano-5-nitro-1,2,3-triazole (**3**). The synthesis route was described in Scheme 1. 4,5-Dicyano-1,2,3-triazole was prepared by one step reaction from diaminomaleodinitrile according to the literature method [26]. 4,5-dicyano-1,2,3-triazole could be hydrolyzed with 1M HCl and 1M acetic acid to obtain 4-carboxamide-5-cyano-1,2,3-triazole (**1**) in 67% yield. Then, 4-cyano-5-amino-1,2,3-triazole (**2**) was synthesized with compound **1** as primary substance via Hofmann rearrangement in 41% yield. The amino group in compound **2** could be oxidized directly with oxidation reagent. Therefore, 4-cyano-5-nitro-1,2,3-triazole (**3**) was synthesized from compound **2** with 30% hydrogen peroxide and 98% sulfuric acid for 24 h. The molecular structures and crystal packing diagrams of compounds **1**·H_2_O and **3**·H_2_O were shown in Figure 1a,b. The detail crystallographic information was summarized in Supplementary Materials. All detailed crystallographic data of the fourteen crystals could be obtained by the link shown in Appendix A. Both compounds, **1**·H_2_O and **3**·H_2_O, crystallize in the monoclinic space group P2(1)/c with four molecules per unit cell (Z = 4). All of them revealed a relative low density value with 1.529 and 1.541 g cm^−3^, respectively, because of the presence of the one water molecule in the crystal structures. As shown in the crystal packing diagram of compound **1**·H_2_O, it can be observed that the H atoms of the formamide group as donors formed hydrogen bonds (N4–H4∙∙∙O1 and N4–H4∙∙∙N1) with the adjacent O atoms from formamide group and N atoms from 1,2,3-triazole ring. Besides, the water molecules were also participated in the formation of hydrogen bonds, such as, N2–H2∙∙∙O2, O2–H2∙∙∙O1, and O2–H2∙∙∙N5. As for compound **3**·H_2_O, the H atoms from the ring and water molecule formed hydrogen bonds (N2–H2∙∙∙O3, O2–H3∙∙∙O1, and O2–H3∙∙∙N4) with the O and N atoms to stabilize the molecular structure.

**Scheme 2:**
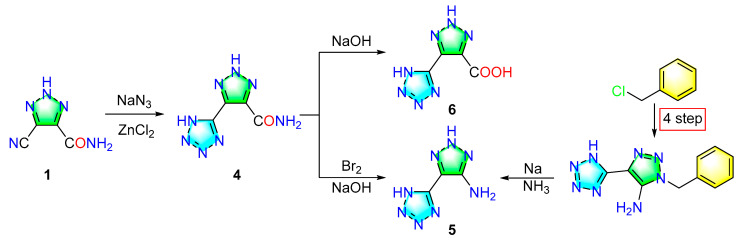
Synthesis of compounds **4**–**6**.

The compound **1** was synthesized according to the above method. Then, the reaction of compound **1** with sodium azide and zinc chloride in a 1,3-dipolar cycloaddition achieved 4-carboxamide-5-(1*H*-tetrazol-5-yl)-1,2,3-triazole (**4**) in 78% yield. 4-amino-5-(1*H*-tetrazol-5-yl)-1,2,3-triazole (**5**) was reported by Dániel Izsák in 2015 [27], in which literature presented the preparation of the nitrogen-rich compound **5** from benzyl chloride in five steps. In the last step, debenzylation was carried out with sodium in liquid ammonia to obtain compound **5**, which had the drawbacks of a complex sequence of reaction steps and associated operations. Here, a new synthetic route was proposed and described in Scheme 2. Compound **5** was prepared from **4** by Hofmann rearrangement. The results of characterization are consistent with the reference [17]. Compound **4** also could be hydrolyzed with sodium hydroxide to obtain 4-carboxylic acid-5-(1*H*-tetrazol-5-yl)-1,2,3-triazole (**6**) in 79% yield. The crystal of compound 4 was characterized by the sodium salt (labeled by **S4**). The molecular structures and packing diagrams of compounds **S4** and **6**·2H_2_O were plotted in Figure 1c,d. The crystal **S4** crystallize in the triclinic space group P-1 with one molecule per unit cell (Z = 1), in which contains one sodium cation, one 4-carboxamide-5-(1*H*-tetrazol-5-yl)-1,2,3-triazolium anion, and two crystal water molecules. Additionally, compound **6**·2H_2_O in the monoclinic space group Pc with two molecules per unit cell (Z = 2) comprising of one 4-carboxylic acid-5-(1*H*-tetrazol-5-yl)-1,2,3-triazole, and two water molecules.

**Scheme 3:**
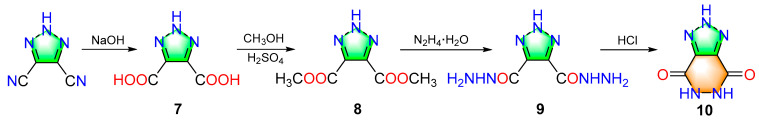
Synthesis of compounds **7**–**10**.

**Figure 2:**
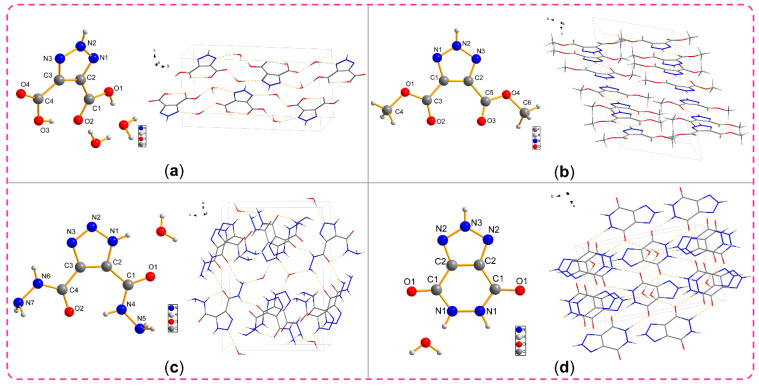
The crystal structures and crystal packing of compounds **7**·2H_2_O (**a**)**, 8** (**b**)**, 9**·H_2_O (**c**)**, and 10**·H_2_O (**d**).

**Scheme 4:**
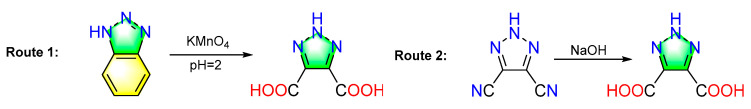
Two synthesis methods of compounds **7**.

As shown in Scheme 3, 4,5-dicyano-1,2,3-triazole was easy to be hydrolyzed under alkaline condition by using sodium hydroxide to achieve 4,5-dicarboxylic acid-1,2,3-triazole (**7**) in 91% yield. Compound **7** was esterified with methanol, concentrated sulfuric acid as a catalyst, to obtain 4,5-dicarboxylic acid dimethyl ester-1,2,3-triazole (**8**) in 83% yield. Subsequently, the 4,5-dicarbohydrazide-1,2,3-triazole (**9**) was synthesized from compound **8** by hydrazinolysis in 86% yield. With hydrochloric acid as catalyst, 4,7-dione-1,2,3-triazolo[4,5-d]pyridazine (**10**) was synthesized from compound **9** taking place condensation reaction in 76% yield. The molecular structures with atomic numbering and crystal packing diagrams of compounds **7**–**10** were placed in Figure 2a–d. Compounds **7**·2H_2_O and **9**·H_2_O crystallize in the monoclinic system space group P2(1)/c with four molecules per unit cell (Z = 4), whereas compound **8** crystallizes in the triclinic space group P-1 and crystal **10**·H_2_O crystallizes in the same crystalline system with **7**·2H_2_O and **9**·H_2_O space group C2/c with four molecular units in a single unit cell. All crystals exhibited lamellar-like stacking mode.

According to the conclusion reported in the literature [28,29], the synthetic method of compound **7** was almost described in Scheme 4. The method in reference has the following disadvantages: (1) Potassium permanganate has a potential explosive risk that concentrated sulfuric acid reacts with KMnO_4_ to give Mn_2_O_7,_ which could be explosive. (2) Manganese dioxide can pollute the environment beyond redemption. (3) The dropping speed of potassium permanganate solution should be controlled during the operation; post-treatment has four steps that include filtration, concentration, acidification, and decolorization. (4) Low yield: 68–75%. Surprisingly, a new synthetic method of **7** was found in the present work. Compared with the literature, the merits of the new method are as follows: (1) Sodium hydroxide presents the lowest explosive risk. (2) Besides aim product, the sodium chloride is formed. Therefore, products have no manganese pollution. (3) The experimental procedure is one-step feeding; post-treatment has only two steps just involving acidification and filtration. (4) High yield: 91%.

**Scheme 5:**
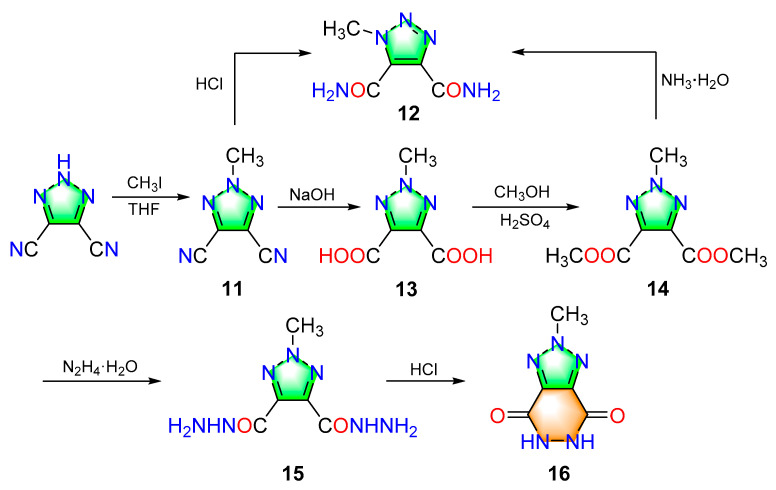
Synthesis of compounds **11**–**16**.

Based on the active hydrogen atom exiting in the triazole ring, we explored the synthesis of methylated derivatives. The synthesis route was introduced in Scheme 5. 2-methyl-4,5-dicyano-1,2,3-triazole (**11**) was successfully formed with iodomethane as a methylation agent in 71% yield. 2-methyl-4,5-dicarboxylic acid-1,2,3-triazole (**13**) was also synthesized from compound **11** via hydrolysis in 89% yield. 2-methyl-4,5-dicarboxylic acid dimethyl ester-1,2,3-Triazole (**14**) was prepared from 13 by esterification in 82% yield. On the one hand, compound **11** could to be hydrolyzed under acidic condition to gain 1-methyl-4,5-dicarboxamide-1,2,3-triazole (**12**) in 62% yield; on the other hand, **14** and concentrated aqueous ammonia reacted for 12 h to produce compound **12**. 2-methyl-4,5-dicarbohydrazide-1,2,3-triazole (**15**) and 2-methyl-4,7-dione-1,2,3-triazolo[4,5-d]pyridazine (**16**) were synthesized according to the previous method which was introduced in Scheme 5.

**Figure 3:**
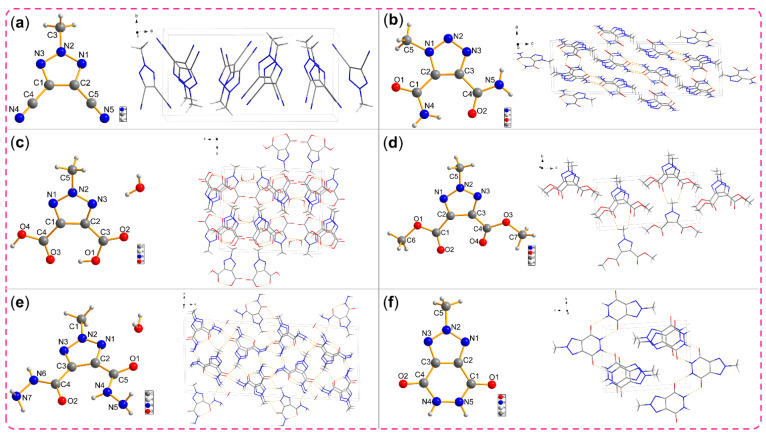
The crystal structures and crystal packing of compounds **11** (**a**), **12** (**b**), **13**·H_2_O (**c**), **14** (**d**), **15**·H_2_O (**e**), and **16** (**f**).

The molecular structures and crystal packing diagrams of crystals **11**–**16** were plotted in Figure 3a–f. From the molecular structures, we can see that all compounds contain no water except for compounds **13**·H_2_O and **15**·H_2_O. Additionally, they belong to different crystalline systems with orthorhombic (**11**), monoclinic (**12**, **13**, **14**, and **15**), and triclinic (**16**), respectively. Obviously, all crystals possessed a plane-layered stacked structure formed by several hydrogen bonds and π-π interactions observed from crystal packing pictures. Notably, the two novel 1,2,3-triazole-fused heterocyclic compounds **10**·H_2_O and **16** had a perfect coplanar geometry, which could be further studied to obtain more 1,2,3-triazole-fused derivatives with good properties, including thermal stability, energetic performance, and low sensitivity.

### 2.2. Thermal Behavior

**Figure 4:**
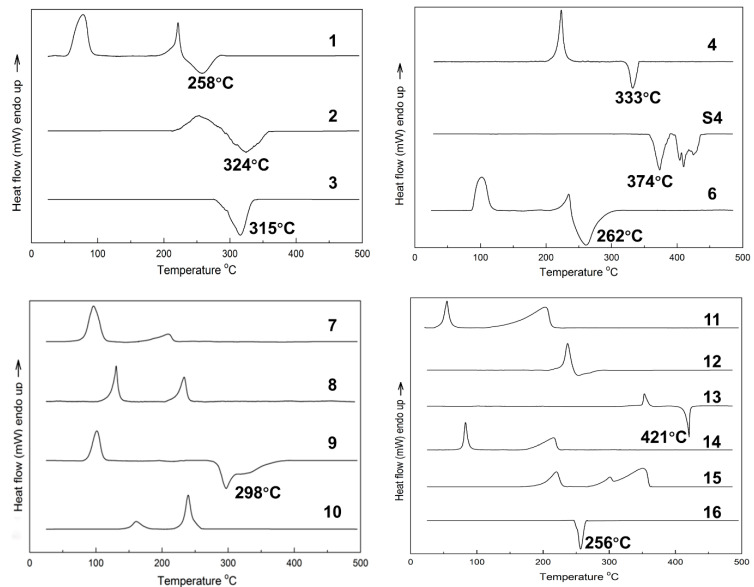
The DSC curves of compounds **1**–**16** recorded at a heating rate of 10 °C·min^−1^.

The thermal stabilities of the newly prepared compounds were evaluated using DSC at a heating rate of 10 °C·min^−1^ under a nitrogen atmosphere. The DSC curves were shown in Figure 4 with the main exothermic decomposition peak temperature (T_d_), and decomposition temperature values are listed in Table 1. Compounds **7**, **8**, **10**, **11**, **14**, and **15** only possessed an endothermic process at the experimental range of 40–500 °C. The exothermic decomposition temperatures of other compounds were in the range of 256 °C (**16**) and 421 °C (**13**). The sodium salt **S4** revealed a multi-stage decomposition progress. As can be seen, most 1,2,3-triazole-based molecules were thermally stable beyond 250 °C (Figure 4) and met the demand of thermal stability for energetic compounds in practical application.

### 2.3. Energetic Properties and Safety

Compounds **3**·H_2_O, **6**·2H_2_O, **10**·H_2_O, and **16** were selected to explore the detonation performance and sensitivity to impact and friction. The crucial parameters were summarized in Table 1. 

Enthalpy of formation is one of the most important parameters to obtain the detonation velocity and pressure for energetic compounds. Thus, the heats of formation for **3**·H_2_O, **6**·2H_2_O, **10**·H_2_O, and **16** were obtained via the atom equivalent scheme by converting quantum mechanical energies of atoms to the enthalpy of formation of the molecule in the gas phase, Equation (1) [30]. The enthalpy of sublimation either vaporization can be predicted through the calculation of molecular electrostatic potentials [31,32], the equation represented as Equation (2). Then, the Hess’ law of constant heat summation was employed to determine enthalpies of formation of the condensed-phase shown in Equation (3) [33]. The geometries of the four compounds were optimized by using the hybrid DFT-B3LYP functional with the 6-311++g(d,p) basis set. All the ab initio calculations of this work were performed via the commercial Gaussian 09 suite of programs [34]. As shown in Table 1, crystals **3**·H_2_O, **6**·2H_2_O, **10**·H_2_O, and **16** exhibited positive enthalpies of formation of 245.0, 204.5, 92.1, and 154.3 kJ·mol^−1^, respectively. Compound **3**·H_2_O had the highest Enthalpy of formation among them, which could be attribute to the introduction of the energetic nitro group.

(1) $$\Delta H_{f}\;\left(g\right)=E\left(g\right)-\Sigma_{i}n_{i}x_{i}$$

(2) $$\Delta H\;\left(sublimation\right)=a{\left(SA\right)}^{2}+b{\left(\upsilon \sigma_{tot}^{2}\right)}^{1/2}+c$$

(3) ΔH (solid)= ΔH (gas)− ΔH (sublimation)

In Equation (2), *SA* is the molecular surface area of the 0.001 electron/bohr^3^ isosurface of the electron density of the molecule, $\sigma_{tot}^{2}$ is described as an indicator of the variability of the electrostatic potential on the molecular surface, and *υ* is interpreted as showing the degree of balance between the positive and negative potentials on the molecular surface where *a*, *b*, and *c* are fitting parameters.

Based on the experimental crystal density values and calculated enthalpies of formation, the detonation properties of the four compounds were estimated by using EXPLO5 (v6.04) program [35]. The energetic parameters were listed in Table 1. Results indicated that the four molecules had moderate detonation parameters, with detonation velocities in the range of 6.9 km·s^−1^ and 7.9 km·s^−1^ and detonation pressures between 16.6 GPa and 23.9 GPa, respectively. Compare the detonation parameters of compounds **10**·H_2_O and **16**, it can be easily observed that the existence of methyl group greatly reduces the density and then decreases the detonation properties of the compound **16**.

The impact and friction sensitivity were tested by employing a standard BAM fall hammer and a BAM friction tester, respectively. All compounds exhibited low mechanical sensitivities (IS: > 40 J; FS: > 360 N). Combined with thermal stability and detonation performance, compound **6**·2H_2_O possessed the most balanced comprehensive properties (*d* = 1.659 g·cm^−3^, P = 23.9 GPa, D = 7.9 km·s^−1^, T_d_ = 262 °C, IS > 40 J, FS > 360 N). Therefore, all these 1,2,3-triazole-based derivatives could be used as precursors to prepare more energetic materials with excellent detonation performance.

## 3. Materials and Methods

### 3.1. General Information

All chemical reagents and solvents of analytical grade were obtained from commercially sources. Elemental analyses were performed on a Flash EA 1112 fully automatic trace element analyzer (Waltham, MA, USA). The FT-IR spectra were recorder as KBr pellets on a Bruker Equinox 55 (Bruker, Germany). Mass spectra were recorded on an Agilent 500-MS (Palo Alto, CA, USA). The single-crystal X-ray diffraction analysis was carried out by on Bruker CCD area-detector diffractometer (Bruker, Germany). The single-crystal X-ray diffraction data were collected at 293–298 K using a Bruker CCD area detector diffractometer equipped with a graphite-monochromatized Mo/Kα radiation (λ = 0.71073 Å) using phi and omega scans. Data collection and initial unit cell refinement were performed with Bruker SMART (Bruker, Germany). Data reduction were performed by using Bruker SHELXTL (Bruker, Germany). Using Olex2 [36], the structures were further solved and refined with the aid of the programs using direct methods and full-matrix least-squares method on F2 by SHELXS–97 and SHELXL–97 [37]. The full-matrix least-squares refinement on F2 involved atomic coordinates and anisotropic thermal parameters for all non-H atoms. The H atoms were included using a riding model. The non-H atoms were refined anisotropically. Differential scanning calorimetry (DSC) was carried out on a model Pyris-1 differential scanning calorimeter under nitrogen atmosphere. Impact and friction sensitivities were performed on a BAM fall hammer BFH-10 and a BAM friction apparatus FSKM-10, respectively.

### 3.2. Synthesis

*4-carboxamide-5-cyano-1,2,3-triazole* (**1**): 4,5-Dicyano-1,2,3-triazole (2.38 g, 20 mmol) was refluxed in an aqueous solution of AcOH (1 M, 25 mL) and hydrochloric acid (1 M, 25 mL) for 30 min. The solution was cooled to 0 °C, whereby the precipitate formed was filtered off and recrystallized from hot water to afford 4-carboxamide-5-cyano-1,2,3-triazole (**1**) hydrate colorless needles (1.84 g, 67%). IR (KBr, cm^−1^): 3545, 3481, 3397, 3305, 3242, 2918, 2850, 2606, 2263, 2151, 1942, 1705, 1612, 1521, 1434, 1366, 1215, 1145, 990, 953, 795, 720, 640. MS (ESI^−^): *m*/*z* = 136.10 ([M]^−^). EA for C_4_H_3_N_5_O (%): calcd: C 35.04, H 2.21; N 51.08; found: C 35.12, H 2.16, N 51.15.

*4-cyano-5-amino-1,2,3-triazole* (**2**): To a solution of NaOH (7.60 g, 0.19 mol) in 60 mL of water, with stirring and cooling (0 °C) was introduced Bromine (2 mL, 0.038 mol). The solution was added 4.38 g (0.032 mol) of 4-carboxamide-5-cyano-1,2,3-triazole (**1**) followed by stirring at 20 °C until full dissolution, after which the reaction mixture was heated for 30 min at 60 °C. The mixture was then cooled and concentrated hydrochloric acid was added to pH ≈ 1. The precipitate was filtered off and washed with cold water (1.43 g, 41%). IR (KBr, cm^−1^): 3394, 3315, 3191, 3052, 2836, 2358, 2246, 1922, 1710, 1601, 1496, 1361, 1284, 1133, 993, 821, 765, 701, 634. MS (ESI^−^): *m*/*z* = 108.10 ([M]^−^). EA For C_3_H_3_N_5_ (%): calcd: C 33.03, H 2.77, N 64.20; found: C 33.38, H 2.81, N 64.03.

*4-cyano-5-nitro-1,2,3-triazole* (**3**): To a mixture of 8 mL of 30% H_2_O_2_ and 15 mL concentrated H_2_SO_4_ was added in small portions, with stirring and cooling (<10 °C). The solution was added 0.85 g (0.0078 mol) of 4-cyano-5-amino-1,2,3-triazole (**2**) and followed by standing for 24 h at room temperature. The reaction mixture was then poured into 60 mL of cold water and extracted with ether (3 × 30 mL). The ether extracts were dried over MgSO_4_. The ether was filtered off and removed (0.55 g, 51%). IR (KBr, cm^−1^): 3533, 3467, 3395, 3309, 3231, 2873, 2850, 2588, 2251, 2131, 1936, 1710, 1605, 1551, 1428, 1379, 1284, 1136, 992, 831, 725, 636. MS (ESI^−^): *m*/*z* = 138.10 ([M]^−^). EA for C_3_HN_5_O_2_ (%): calcd: C 25.91, H 0.72, N 50.36; found: C 25.83, H 0.77, N 50.43.

*4-carboxamide-5-(1H-tetrazol-5-yl)-1,2,3-triazole* (**4**): 4-carboxamide-5-cyano-1,2,3-triazole (**1**, 1.37g 10 mmol) was dissolved in water (50 mL) and sodium azide (0.78 g, 12 mmol) and zinc chloride (1.5 g, 11 mmol) were added. The reaction mixture was stirred and heated under reflux for 4 h. The solution was then cooled to ambient temperature and concentrated hydrochloric acid (10 mL) was added while cooling to 0 °C. The precipitate was removed by filtration under suction and the product was washed with cold water and ethanol to obtain 4-carboxamide-5-(1H-tetrazol-5-yl)-1,2,3-triazole (4, 1.4g 78%). IR (KBr, cm^−1^): 3348, 3223, 3092, 2921, 2858, 2781, 1685, 1587, 1469, 1309, 1217, 1175, 1125, 1048, 992, 858, 714, 666. MS (ESI^−^): *m*/*z* = 179.10 ([M]^−^). EA for C_4_H_4_N_8_O (%): calcd: C 26.67, H 2.24; N 62.21; found: C 26.73, H 2.28, N 62.17.

*4-amino-5-(1H-tetrazol-5-yl)-1,2,3-triazole* (**5**): Like the synthetic method of compound **2**. To a solution of NaOH (7.60 g, 0.19 mol) in 60 mL of water, with stirring and cooling (0 °C) was introduced Bromine (2 mL, 0.038 mol). The solution was added 5.76 g (0.032 mol) of 4-carboxamide-5-(1*H*-tetrazol-5-yl)-1,2,3-triazole (**4**) followed by stirring at 20 °C until full dissolution, after which the reaction mixture was heated for 30 min at 60 °C. The mixture was then cooled and concentrated hydrochloric acid was added to pH ≈ 1. The precipitate was filtered off and washed with cold water (2.31 g, 40 %). IR (KBr, cm^−1^): 3435, 3318, 3227, 3036, 2976, 2893, 2794, 2553, 1626, 1508, 1411, 1372, 1214, 1183, 1075, 971, 866, 719, 691. MS (ESI^−^): *m*/*z* = 151.10 ([M]^−^). EA for C_3_H_4_N_8_ (%): calcd: C 23.69, H 2.65; N 73.66; found: C 23.85, H 2.61, N 73.57

*4-carboxylic acid-5-(1H-tetrazol-5-yl)-1,2,3-triazole* (**6**): 4-carboxamide-5-(1*H*-tetrazol-5-yl)-1,2,3-triazole (**4**, 1.80 g, 0.01 mol) was refluxed in water (20 mL) and NaOH (1.60 g, 0.04 mol) for 4 h. The solution was cooled and concentrated hydrochloric acid was added to pH ≈ 1. The precipitate was filtered off and washed with cold water (1.43 g, 79 %). IR (KBr, cm^−1^): 3390, 3261, 3142, 3053, 2918, 2779, 2632, 1646, 1509, 1320, 1150, 1063, 989, 845, 735, 662. MS (ESI^−^): *m*/*z* = 180.10 ([M]^−^). EA for C_4_H_3_N_7_O_2_ (%): calcd: C 26.53, H 1.67; N 54.14; found: C 26.58, H 1.62, N 54.22.

*4,5-dicarboxylic acid-1,2,3-triazole* (**7**): 4,5-Dicyano-1,2,3-triazole (11.90 g, 0.1 mol) was refluxed in water (100 mL) and NaOH (20.00 g, 0.5 mol) for 5h. The solution was cooled and concentrated hydrochloric acid was added to pH ≈ 1. The reaction mixture was stirred for 45 min and cooled to 0 °C. The precipitate was filtered off and washed with cold water (14.3 g, 91 %). IR (KBr, cm^−1^): 3462, 3213, 2918, 2850, 2428, 2264, 1934, 1711, 1606, 1528, 1488, 1385, 1200, 999, 832, 773, 687. MS (ESI^−^): *m*/*z* = 156.10 ([M]^−^). EA for C_4_H_3_N_3_O_4_ (%): calcd: C 30.58, H 1.93, N 26.75; found: C 30.52, H 1.97, N 26.81.

*4,5-dicarboxylic acid dimethyl ester-1,2,3-Triazole* (**8**): 4,5-dicarboxylic acid-1,2,3-triazole (**7**, 11.90 g, 0.1 mol) was refluxed in absolute methanol (200 mL) and 98% H_2_SO_4_ (20 mL) for 5 h. The solution was cooled and NaHCO_3_ was added to neutralize H_2_SO_4_, then filtered. The methanol was allowed to evaporate and water was added (150 mL). The mixture extracted with ethyl acetate (3 × 200 mL). The ethyl acetate was removed in the rotary evaporator and to yield a white powder (12.28 g, 83 %). IR (KBr, cm^−1^): 3239, 3108, 2966, 2852, 2558, 2029, 1740, 1523, 1438, 1387, 1302, 1231, 1190, 990, 949, 836, 806, 771, 676. MS (ESI^−^): *m*/*z* = 184.10 ([M]^−^). EA for C_6_H_7_N_3_O_4_ (%): calcd: C 38.93, H 3.81, N 22.70; found: C 38.89, H 3.85, N 22.73.

*4,5-dicarbohydrazide-1,2,3-Triazole* (**9**): To a stirred solution of 9.25 g (0.05 mol) of 4,5-dicarboxylic acid dimethyl ester-1,2,3-Triazole (**8**) in absolute methanol (250 mL), 32 mL of 80 % hydrazine hydrate was added dropwise. The mixture was refluxed for 5 h. After cooling to 0 °C, the precipitate was suspended in 150 mL of water and acidified to pH 3 ~ 4 with 2 M hydrochloric acid. After stirring for 30 min, the precipitate was filtered off and washed with ethanol and ether (7.96 g, 86 %). IR (KBr, cm^−1^): 3497, 3288, 3007, 2676, 1922, 1674, 1604, 1559, 1524, 1387, 1319, 1259, 1213, 1125, 1045, 989, 961, 893, 804, 782, 736, 661. MS (ESI^−^): *m*/*z* = 184.10 ([M]^−^). EA for C_4_H_7_N_7_O_2_ (%): calcd: C 25.95, H 3.81, N 52.96; found: C 25.98, H 3.84, N 52.92.

*4,7-dione-1,2,3-triazolo[4,5-d]pyridazine* (**10**): 4,5-dicarbohydrazide-1,2,3-Triazole (9, 3.70 g, 0.02 mol) was refluxed in concentrated hydrochloric acid (3 M, 30 mL) for 4 h. The solution was cooled to 0 °C, whereby the precipitate formed was filtered off and washed with cold water (2.3 g, 76 %). IR (KBr, cm^−1^): 3416, 3313, 3075, 2919, 2811, 2655, 1944, 1709, 1639, 1499, 1418, 1372, 1225, 1114, 1006, 976, 851, 811, 777, 690. MS (ESI^−^): *m*/*z* = 151.95 ([M]^−^). EA for C_4_H_3_N_5_O_2_ (%): calcd: C 31.38, H 1.98, N 45.74; found: C 31.44, H 1.96, N 45.79.

*2-methyl-4,5-dicyano-1,2,3-triazole* (**11**): The 4,5-dicyano-1,2,3-triazole (7.14 g, 0.06 mol) was dissolved in tetrahydrofuran (120 mL) and the anhydrous potassium carbonate was added. The mixture was stirred until no bubbles were formed in the flask. At low temperatures (< 5 °C), the iodomethane (5.6 mL, 0.09 mol) was added. The mixture was stirred for 24 h at room temperature. The tetrahydrofuran was allowed to evaporate and water was added (150 mL). The mixture extracted with ether (3 × 200 mL). The ethyl acetate was removed to yield yellow crystal (5.67 g, 71 %). IR (KBr, cm^−1^): 3513, 3420, 3368, 3248, 3160, 2958, 2843, 2633, 2263, 2143, 1973, 1682, 1526, 1441, 1225, 1190, 985, 951, 783, 718. MS (ESI^−^): *m*/*z* = 132.10 ([M]^−^). EA for C_5_H_3_N_5_ (%): calcd: C 45.12, H 2.27, N 52.61; found: C 45.18, H 2.23, N 52.65.

*1-methyl-4,5-dicarboxamide-1,2,3-triazole* (**12**): Method 1: 2-methyl-4,5-dicyano-1,2,3-triazole (**11**, 1.33 g, 0.01 mol) was refluxed in solution of AcOH (3 M, 12 mL) and hydrochloric acid (3 M, 12 mL) for 1 h. The solution was cooled to 0 °C, whereby the precipitate was filtered off and recrystallized from hot water to afford colorless crystal (1.04 g, 62%). Method 2: 2-methyl-4,5-dicarboxylic acid dimethyl ester-1,2,3-Triazole (**14**, 1 g, 0.005 mol) was stirred in ammonium hydroxide (10 mL) at room temperature for 12 h. The excess of ammonium hydroxide was removed in the rotary evaporator and concentrated hydrochloric acid was added dropwise to bring the residue to pH ≈ 1. The precipitate was filtered off, washed with cold water (0.57 g, 68 %). IR (KBr, cm^−1^): 3462, 3357, 3238, 3174, 2872, 2602, 2178, 1938, 1664, 1590, 1487, 1380, 1217, 1125, 996, 757, 640. MS (ESI^−^): *m*/*z* = 168.10 ([M]^−^). EA for C_5_H_7_N_5_O_2_ (%): calcd: C 35.51, H 4.17, N 41.41; found: C 35.54, H 4.13, N 41.45.

*2-methyl-4,5-dicarboxylic acid-1,2,3-triazole* (**13**): Similar to the synthetic method of compound **7**. 2-methyl-4,5-dicyano-1,2,3-triazole (**11**, 3.99 g, 0.03 mol) was refluxed in water (30 mL) and NaOH (6.00 g, 0.15 mol) for 6h. The solution was cooled and concentrated hydrochloric acid was added to pH ≈ 1. The reaction mixture was stirred for 45 min and cooled to 0 °C. The precipitate was filtered off and washed with cold water (4.57 g, 89%). IR (KBr, cm^−1^): 3443, 3231, 2850, 2448, 2252, 1713, 1604, 1535, 1479, 1381, 1288, 1216, 990, 828, 770, 681, 652. MS (ESI^−^): *m*/*z* = 169.95 ([M]^−^). EA for C_5_H_5_N_3_O_4_ (%): calcd: C 35.10, H 2.95, N 24.56; found: C 35.14, H 2.92, N 24.59.

*2-methyl-4,5-dicarboxylic acid dimethyl ester-1,2,3-Triazole* (**14**): Similar to the synthetic method of compound **8**. 2-methyl-4,5-dicarboxylic acid-1,2,3-triazole (**13**, 3.42 g, 0.02 mol) was refluxed in absolute methanol (60 mL) and 98% H_2_SO_4_ (6 mL) for 6 h. The solution was cooled and NaHCO_3_ was added to neutralize H_2_SO_4_, then filtered. The methanol was removed in the rotary evaporator. The precipitate was washed with cold water (3.26 g, 82%). IR (KBr, cm^−1^): 3226, 3102, 2863, 2541, 2029, 1736, 1525, 1438, 1383, 1316, 1230, 1187, 986, 940, 811, 770, 672. MS (ESI^−^): *m*/*z* = 198.10 ([M]^−^). EA C_7_H_9_N_3_O_4_ (%): calcd: C 42.21, H 4.55, N 21.10; found: C 42.25, H 4.52, N 21.13.

*2-methyl-4,5-dicarbohydrazide-1,2,3-triazole* (**15**): To a stirred solution of 4.00 g (0.02 mol) of 2-methyl-4,5-dicarboxylic acid dimethyl ester-1,2,3-Triazole (**14**) in absolute methanol (80 mL), 13 mL of 80 % hydrazine hydrate was added dropwise. The mixture was refluxed for 5 h. After cooling to 0 °C, the precipitate was filtered off and washed with cold water and ethanol (3.46 g, 87 %). IR (KBr, cm^−1^): 3487, 3293, 3011, 2655, 1932, 1602, 1544, 1380, 1312, 1236, 1125, 1047, 980, 962, 802, 783, 661. MS (ESI^−^): *m*/*z* = 198.10 ([M]^−^). EA for C_5_H_9_N_7_O_2_ (%): calcd: C 30.15, H 4.55, N 49.23; found: C 30.12, H 4.59, N 49.26.

*2-methyl-4,7-dione-1,2,3-triazolo[4,5-d]pyridazine* (**16**): Similar to the synthetic method of compound **10**. 2-methyl-4,5-dicarbohydrazide-1,2,3-triazole (**15**, 1.99 g, 0.01 mol) was refluxed in concentrated hydrochloric acid (3 M, 10 mL) for 5 h. The solution was cooled to 0 °C, whereby the precipitate formed was filtered off and washed with cold water (1.24 g, 74 %). IR (KBr, cm^−1^): 3416, 3317, 3081, 2976, 2805, 2643, 1961, 1702, 1622, 1510, 1409, 1381, 1327, 1219, 1110, 976, 851, 775, 690. MS (ESI^−^): *m*/*z* = 166.10 ([M]^−^). EA for C_5_H_5_N_5_O_2_ (%): calcd: C 35.93, H 3.02, N 41.91; found: C 35.91, H 3.06, N 41.95.

## 4. Conclusions

In summary, we have shown a study on the 1,2,3-triazole-based compounds and designed five routes to synthesize fifteen compounds starting from commercially available diaminomaleonitrile. The operation of experiments is simple, convenient, and sustainable. All prepared compounds were characterized by EA, FT-IR, and MS spectra. Additionally, the crystal structure of fourteen compounds were cultivated and determined with single crystal X-ray crystallography technology. Among them, nine compounds have high nitrogen content (compound **1**, N% = 51%; **2**, N% = 64%; **3**, N% = 50%; **4**, N% = 62%; **6**, N% = 54%; **9**, N% = 53%; **10**, N% = 46%; **15**, N% = 49%; **16**, N% = 42%), of which as high-nitrogen content molecules has a potential possibility to be used in pyrotechnic fire extinguishers as a coolant and reductant, which can produce large quantities of nitrogen when combusted. The DSC results indicated that these compounds had good thermal stabilities with the exothermic decomposition temperatures between 256 °C (**16**) to 421 °C (**13**), except for the six compounds **7**, **8**, **10**, **11**, **14**, and **15**. Four compounds **3**·H_2_O, **6**·2H_2_O, **10**·H_2_O, and **16** were selected to explore the detonation performance and sensitivity. We found that the four compounds exhibited a moderate detonation performance, with detonation velocities in the range of 6.9 km·s^−1^ and 7.9 km·s^−1^ and detonation pressures between 16.6 GPa and 23.9 GPa, respectively. As expected, they are also insensitive to impact and friction (IS: > 40 J; FS: > 360 N). Consequently, these compounds have the potential to be a core structure in a brand-new family of explosives with nitro-, nitramino-, and amino-substituents. Additionally, all these compounds could lead to abundant neutral molecules, energetic salts, and complex compounds. The possibilities mentioned above endorse to continue research on 1,2,3-triazole-based derivatives in the field of energetic materials.

## Figures and Tables

**Table 1:** Properties of compounds **3**, **6**, **10**, and **16**.

NO.	Formula	*d*^a^ [g·cm^−3^]	ΔH_f_ ^b^ [kJ·mol^−1^]	P ^c^ [GPa]	D ^d^ [km·s^−1^]	T_d_ ^e^ [°C]	IS ^f^ [J]	FS ^g^ [N]
**3**·H_2_O	C_3_H_3_N_5_O_3_	1.541	245.0	21.2	7.4	315	>40	>360
**6**·2H_2_O	C_4_H_7_N_7_O_4_	1.659	204.5	23.9	7.9	262	>40	>360
**10**·H_2_O	C_4_H_5_N_5_O_3_	1.716	92.1	21.3	7.5	-	>40	>360
**16**	C_5_H_5_N_5_O_2_	1.686	154.3	16.6	6.9	256	>40	>360
TNT	C_7_H_5_N_3_O_6_	1.650	−67.0	19.5	6.9	295	15	353

^a^ Density. ^b^ Enthalpy of formation. ^c^ Detonation pressure. ^d^ Detonation velocity. ^e^ Thermal decomposition temperature under nitrogen. ^f^ Impact sensitivity. ^g^ Friction sensitivity.

## Data Availability

The data presented in this study are available in the paper.

## Supplementary Materials

The following are available online. Figures S1–S14: Crystal structure of **1**, **3**, **4**, **6**, **7**–**10**, **11**–**16**. Table S1 Crystal data and structure refinement for compounds **1** and **3**; Table S2 Crystal data and structure refinement for compounds **4** and **6**; Table S3 Crystal data and structure refinement for compounds **7**~**10**; Table S4 Crystal data and structure refinement for compounds **11**~**16**.

## Sample Availability

Samples of the compounds **1–16** are available from the authors.

## Appendix A

CCDC 1497228, 1879824, 1497227, 1879825, 1879826, 1879827, 1879828, 1879817, 1879818, 1879819, 1879820, 1879821, 1879822, and 1879823 contain the supplementary crystallographic data for this paper. These data can be obtained free of charge via http://www.ccdc.cam.ac.uk/conts/retrieving.html (or from the Cambridge Crystallographic Data Centre, 12, Union Road, Cambridge CB2 1EZ, UK; Fax: +44-1223-336033).
