# Supplementary material for: 4,5-Dicyano-1,2,3-Triazole—A Promising Precursor for a New Family of Energetic Compounds and Its Nitrogen-Rich Derivatives: Synthesis and Crystal Structures
Source: Molecules. 2021 Nov 7;26(21):6735. doi: 10.3390/molecules26216735 (PMC8588547; doi:10.3390/molecules26216735)

checkCIF/PLATON report

You have not supplied any structure factors. As a result the full set of tests cannot be run.

THIS REPORT IS FOR GUIDANCE ONLY. IF USED AS PART OF A REVIEW PROCEDURE FOR PUBLICATION, IT SHOULD NOT REPLACE THE EXPERTISE OF AN EXPERIENCED CRYSTALLOGRAPHIC REFEREE.

No syntax errors found. CIF dictionary Interpreting this report

Datablock: 1_a

```
Bond precision:  C-C = 0.0030 A          Wavelength=0.71073

Cell:            a=13.605(2)     b=3.8339(6)        c=32.858(5)
                 alpha=90        beta=93.499(4)     gamma=90
Temperature:     296 K
```

	Calculated	Reported
Volume	1710.7(5)	1710.7(5)
Space group	P 2/c	P 2/c
Hall group	-P 2yc	-P 2yc
Moiety formula	4(C5 H9 N7 O2), H2 O	?
Sum formula	C20 H38 N28 O9	C20 H38 N28 O9
Mr	814.78	814.78
Dx,g cm-3	1.582	1.582
Z	2	2
Mu (mm-1)	0.128	0.128
F000	852.0	852.0
F000’	852.32	
h,k,lmax	16,4,39	16,4,39
Nref	3035	3027
Tmin,Tmax	0.972,0.977	0.864,0.864
Tmin’	0.972	

```
Correction method= # Reported T Limits: Tmin=0.864 Tmax=0.864
AbsCorr = MULTI-SCAN

Data completeness= 0.997          Theta(max)= 24.998

R(reflections)= 0.0499( 2108)     wR2(reflections)= 0.1421( 3027)

S = 0.973                  Npar= 299
```

The following ALERTS were generated. Each ALERT has the format
test-name_ALERT_alert-type_alert-level.
Click on the hyperlinks for more details of the test.

Alert level C

```
PLAT250_ALERT_2_C Large U3/U1 Ratio for Average U(i,j) Tensor ....        2.1 Note
PLAT250_ALERT_2_C Large U3/U1 Ratio for Average U(i,j) Tensor ....        2.3 Note
PLAT420_ALERT_2_C D-H Without Acceptor       N14    --  H9N    ...     Please Check
PLAT420_ALERT_2_C D-H Without Acceptor       N12    --  H11N   ...     Please Check
PLAT480_ALERT_4_C Long H...A H-Bond Reported H2N    ..  O1      ..       2.63 Ang.
```

Alert level G

```
PLAT002_ALERT_2_G Number of Distance or Angle Restraints on AtSite         21 Note
PLAT172_ALERT_4_G The CIF-Embedded .res File Contains DFIX Records          3 Report
PLAT860_ALERT_3_G Number of Least-Squares Restraints .............         12 Note
```

---

0 **ALERT level A** = Most likely a serious problem - resolve or explain
0 **ALERT level B** = A potentially serious problem, consider carefully
5 **ALERT level C** = Check. Ensure it is not caused by an omission or oversight
3 **ALERT level G** = General information/check it is not something unexpected

0 ALERT type 1 CIF construction/syntax error, inconsistent or missing data
5 ALERT type 2 Indicator that the structure model may be wrong or deficient
1 ALERT type 3 Indicator that the structure quality may be low
2 ALERT type 4 Improvement, methodology, query or suggestion
0 ALERT type 5 Informative message, check

It is advisable to attempt to resolve as many as possible of the alerts in all categories. Often the minor alerts point to easily fixed oversights, errors and omissions in your CIF or refinement strategy, so attention to these fine details can be worthwhile. In order to resolve some of the more serious problems it may be necessary to carry out additional measurements or structure refinements. However, the purpose of your study may justify the reported deviations and the more serious of these should normally be commented upon in the discussion or experimental section of a paper or in the "special_details" fields of the CIF. checkCIF was carefully designed to identify outliers and unusual parameters, but every test has its limitations and alerts that are not important in a particular case may appear. Conversely, the absence of alerts does not guarantee there are no aspects of the results needing attention. It is up to the individual to critically assess their own results and, if necessary, seek expert advice.

Publication of your CIF in IUCr journals

A basic structural check has been run on your CIF. These basic checks will be run on all CIFs submitted for publication in IUCr journals (*Acta Crystallographica*, *Journal of Applied Crystallography*, *Journal of Synchrotron Radiation*); however, if you intend to submit to *Acta Crystallographica Section C* or *E* or *IUCrData*, you should make sure that full publication checks are run on the final version of your CIF prior to submission.

Publication of your CIF in other journals

Please refer to the *Notes for Authors* of the relevant journal for any special instructions relating to CIF submission.

PLATON version of 24/11/2016; check.def file version of 23/11/2016

Datablock 1_a - ellipsoid plot

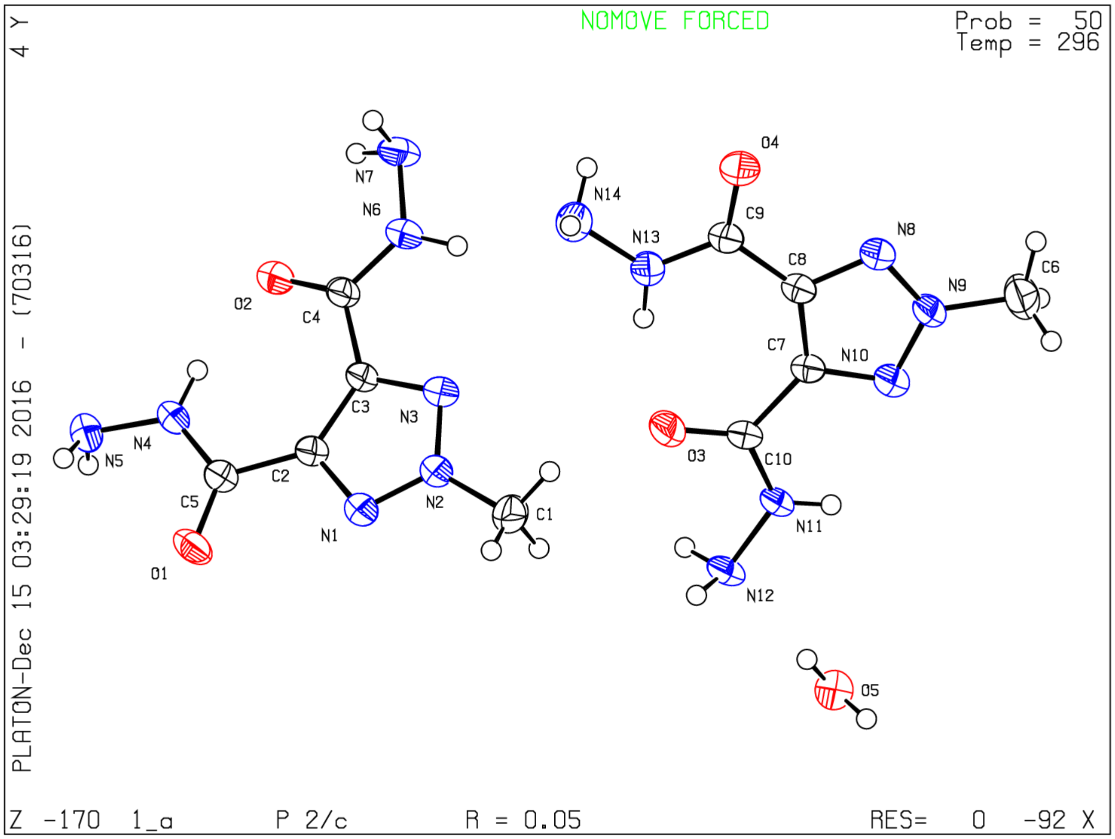

Supplement: Supplementary file 1 [file molecules-26-06735-s001.zip › cif files&checkcif report/checkcif-15.pdf]
