# Supplementary material for: 4,5-Dicyano-1,2,3-Triazole—A Promising Precursor for a New Family of Energetic Compounds and Its Nitrogen-Rich Derivatives: Synthesis and Crystal Structures
Source: Molecules. 2021 Nov 7;26(21):6735. doi: 10.3390/molecules26216735 (PMC8588547; doi:10.3390/molecules26216735)

checkCIF/PLATON report

You have not supplied any structure factors. As a result the full set of tests cannot be run.

THIS REPORT IS FOR GUIDANCE ONLY. IF USED AS PART OF A REVIEW PROCEDURE FOR PUBLICATION, IT SHOULD NOT REPLACE THE EXPERTISE OF AN EXPERIENCED CRYSTALLOGRAPHIC REFEREE.

No syntax errors found. CIF dictionary Interpreting this report

Datablock: 170108c

```
Bond precision:  C-C = 0.0080 A          Wavelength=0.71073

Cell:          a=3.8300(3)       b=8.1261(7)      c=10.8879(9)
               alpha=94.125(1)   beta=99.488(2)   gamma=98.190(2)
Temperature:   298 K

                Calculated                Reported
Volume          329.24(5)                 329.24(5)
Space group     P -1                      P-1
Hall group      -P 1                      ?
Moiety formula  C5 H5 N5 O2               ?
Sum formula     C5 H5 N5 O2               C5 H5 N5 O2
Mr              167.14                    167.14
Dx,g cm-3       1.686                     1.686
Z               2                         2
Mu (mm-1)       0.136                     0.136
F000            172.0                     172.0
F000'           172.07
h,k,lmax        4,9,12                    4,9,12
Nref            1171                      1152
Tmin,Tmax       0.977,0.986               0.972,0.987
Tmin'           0.972

Correction method= # Reported T Limits: Tmin=0.972 Tmax=0.987
AbsCorr = MULTI-SCAN

Data completeness= 0.984          Theta(max)= 25.020

R(reflections)= 0.0969( 862)      wR2(reflections)= 0.3103( 1152)

S = 1.211                  Npar= 110
```

The following ALERTS were generated. Each ALERT has the format
test-name_ALERT_alert-type_alert-level.
Click on the hyperlinks for more details of the test.

Alert level C

```
PLAT084_ALERT_3_C High wR2 Value (i.e. > 0.25) ...................       0.31 Report
PLAT250_ALERT_2_C Large U3/U1 Ratio for Average U(i,j) Tensor ....        2.3 Note
PLAT340_ALERT_3_C Low Bond Precision on  C-C Bonds ...............      0.008 Ang.
```

Alert level G

```
PLAT005_ALERT_5_G No Embedded Refinement Details found  in the CIF     Please Do !
PLAT007_ALERT_5_G Number of Unrefined Donor-H Atoms ..............          2 Report
PLAT066_ALERT_1_G Predicted and Reported Tmin&Tmax Range Identical          ? Check
PLAT072_ALERT_2_G SHELXL First  Parameter in WGHT  Unusually Large       0.13 Report
PLAT093_ALERT_1_G No s.u.'s on H-positions, Refinement Reported as      mixed Check
PLAT899_ALERT_4_G SHELXL97   is Deprecated and Succeeded by SHELXL       2014 Note
```

```
   0 ALERT level A = Most likely a serious problem - resolve or explain
   0 ALERT level B = A potentially serious problem, consider carefully
   3 ALERT level C = Check. Ensure it is not caused by an omission or oversight
   6 ALERT level G = General information/check it is not something unexpected

   2 ALERT type 1 CIF construction/syntax error, inconsistent or missing data
   2 ALERT type 2 Indicator that the structure model may be wrong or deficient
   2 ALERT type 3 Indicator that the structure quality may be low
   1 ALERT type 4 Improvement, methodology, query or suggestion
   2 ALERT type 5 Informative message, check
```

It is advisable to attempt to resolve as many as possible of the alerts in all categories. Often the minor alerts point to easily fixed oversights, errors and omissions in your CIF or refinement strategy, so attention to these fine details can be worthwhile. In order to resolve some of the more serious problems it may be necessary to carry out additional measurements or structure refinements. However, the purpose of your study may justify the reported deviations and the more serious of these should normally be commented upon in the discussion or experimental section of a paper or in the "special_details" fields of the CIF. checkCIF was carefully designed to identify outliers and unusual parameters, but every test has its limitations and alerts that are not important in a particular case may appear. Conversely, the absence of alerts does not guarantee there are no aspects of the results needing attention. It is up to the individual to critically assess their own results and, if necessary, seek expert advice.

Publication of your CIF in IUCr journals

A basic structural check has been run on your CIF. These basic checks will be run on all CIFs submitted for publication in IUCr journals (*Acta Crystallographica*, *Journal of Applied Crystallography*, *Journal of Synchrotron Radiation*); however, if you intend to submit to *Acta Crystallographica Section C* or *E* or *IUCrData*, you should make sure that full publication checks are run on the final version of your CIF prior to submission.

Publication of your CIF in other journals

Please refer to the *Notes for Authors* of the relevant journal for any special instructions relating to CIF submission.

PLATON version of 24/11/2016; check.def file version of 23/11/2016

Datablock 170108c - ellipsoid plot

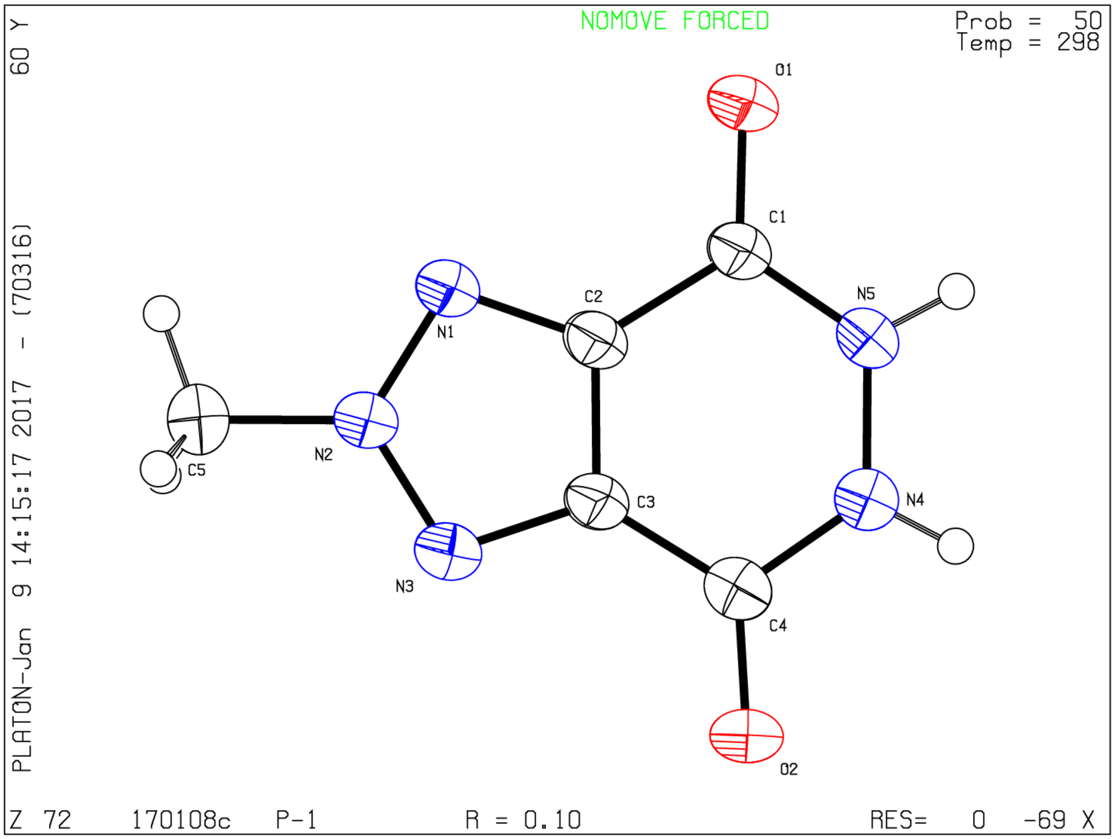

Supplement: Supplementary file 1 [file molecules-26-06735-s001.zip › cif files&checkcif report/checkcif-170108c.pdf]
