# Supplementary material for: 4,5-Dicyano-1,2,3-Triazole—A Promising Precursor for a New Family of Energetic Compounds and Its Nitrogen-Rich Derivatives: Synthesis and Crystal Structures
Source: Molecules. 2021 Nov 7;26(21):6735. doi: 10.3390/molecules26216735 (PMC8588547; doi:10.3390/molecules26216735)
Supplement: Supplementary file 1 [file molecules-26-06735-s001.zip › Supporting Information.pdf]

Supporting Information

4,5-Dicyano-1,2,3-Triazole—A Promising Precursor for a New Family of Energetic Compounds and Its Nitrogen-rich Derivatives: Synthesis and Crystal Structures

Wen-Li Cao[1], Jian Qin[1,2], Jian-Guo Zhang[1,*]

[1] State Key Laboratory of Explosion Science and Technology, Beijing Institute of Technology, Beijing 100081, China.

[2] China North Chemical Research Academy Group CO., LTD., Beijing 100089, China

Table of Contents

X-ray Crystallographic Data 1

Table S1 Crystal data and structure refinement for compounds 1 and 3 1

Table S2 Crystal data and structure refinement for compounds 4 and 6 3

Table S3 Crystal data and structure refinement for compounds 7 ~ 10 5

Table S4 Crystal data and structure refinement for compounds 11 ~ 16 8

X-ray Crystallographic Data

Table S1. Crystal data and structure refinement for compounds 1 and 3.

	**1**	**3**
Empirical formula	$C_4H_5N_5O_2$	$C_3H_3N_5O_3$
MW (g mol^{-1})	155.13	157.10
Temperature (K)	298(2)	293(2)
Crystal System	Monoclinic	Monoclinic
Space Group	P2(1)/c	P2(1)/c
a (Å)	3.6672(3)	3.6790(3)
b (Å)	16.3613(14)	16.3723(14)
c (Å)	11.3345(8)	11.3442(11)
α (°)	90.00	90
β (°)	97.7600(10)	97.785(2)
γ (°)	90.00	90
V (Å)^3	673.84(9)	677.01(10)
Z	4	4
ρ_{calcd} (g cm^{-3})	1.529	1.541
μ (mm^{-1})	0.126	1.210
F (000)	320	320
θ (°)	3.08-25.01	4.77 – 66.35
Reflections collected	3230	2046
Unique reflections	1183	1196
R_{int}	0.0809	0.0603
Data/restraints/parameters	1183 / 0 / 100	1196 / 0 / 100
GOF on F^2	1.018	1.057
R_1, wR_2 [$I>2\sigma(I)$]	R_1=0.0556, wR_2=0.1325	R_1=0.0768, wR_2=0.1684
R_1, wR_2 (all data)	R_1=0.0894, wR_2=0.1491	R_1=0.1605, wR_2=0.2247
CCDC	1497228	1879824

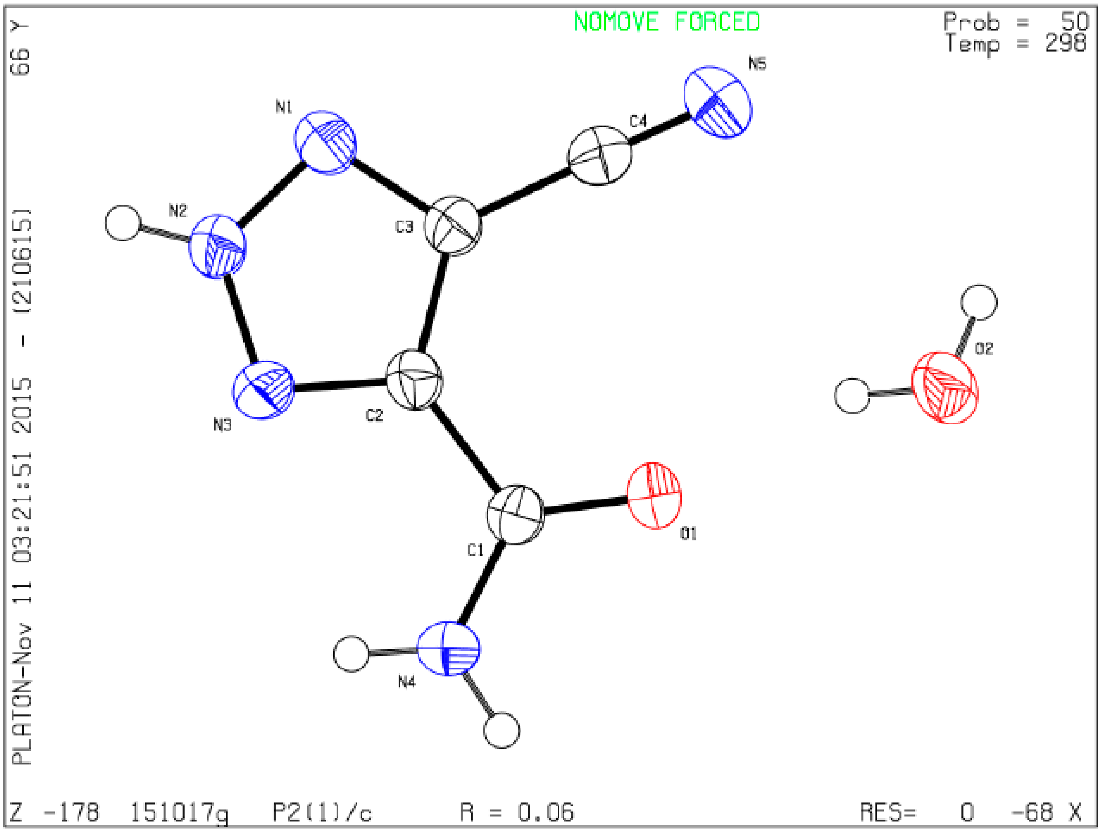

Figure S1. crystal structure of **1.**

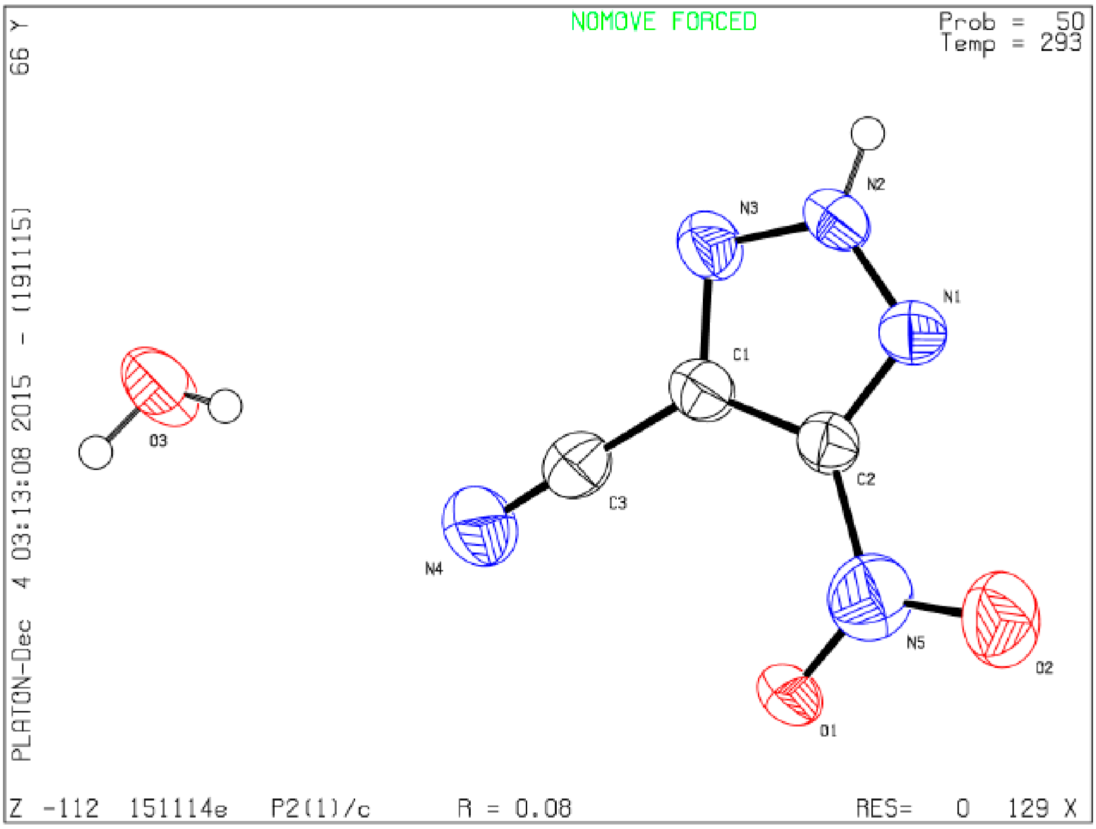

Figure S2. crystal structure of **3.**

Table S2. Crystal data and structure refinement for compounds 4 and 6.

	**Na salt (S4)**	**6**
Empirical formula	$C_8H_{14}N_{16}Na_2O_6$	$C_4H_7N_7O_4$
MW (g mol^{-1})	476.33	217.17
Temperature (K)	298(2)	298(2)
Crystal System	Triclinic	Monoclinic
Space Group	P-1	Pc
a (Å)	6.7660(6)	4.9355(4)
b (Å)	7.2849(7)	5.1873(5)
c (Å)	10.7541(9)	16.9874(14)
α (°)	109.069(2)	90
β (°)	98.9320(10)	90.9210(10)
γ (°)	103.404(2)	90
V (Å)^3	471.70(7)	434.85(7)
Z	1	2
ρ_{calcd} (g cm^{-3})	1.677	1.659
μ (mm^{-1})	0.178	0.146
F (000)	244	224
θ (°)	3.01-25.02	2.40 - 24.98
Reflections collected	1631	2081
Unique reflections	1631	1143
R_{int}	0.0000	0.0442
Data/restraints/parameters	1631 / 0 / 147	1143 / 2 / 136
GOF on F^2	1.042	1.089
R_1, wR_2 [$I>2\sigma(I)$]	R_1=0.0676, wR_2=0.1680	R_1=0.0418, wR_2=0.0946
R_1, wR_2 (all data)	R_1=0.0901, wR_2=0.1811	R_1=0.0523, wR_2=0.1020
CCDC	1497227	1879825

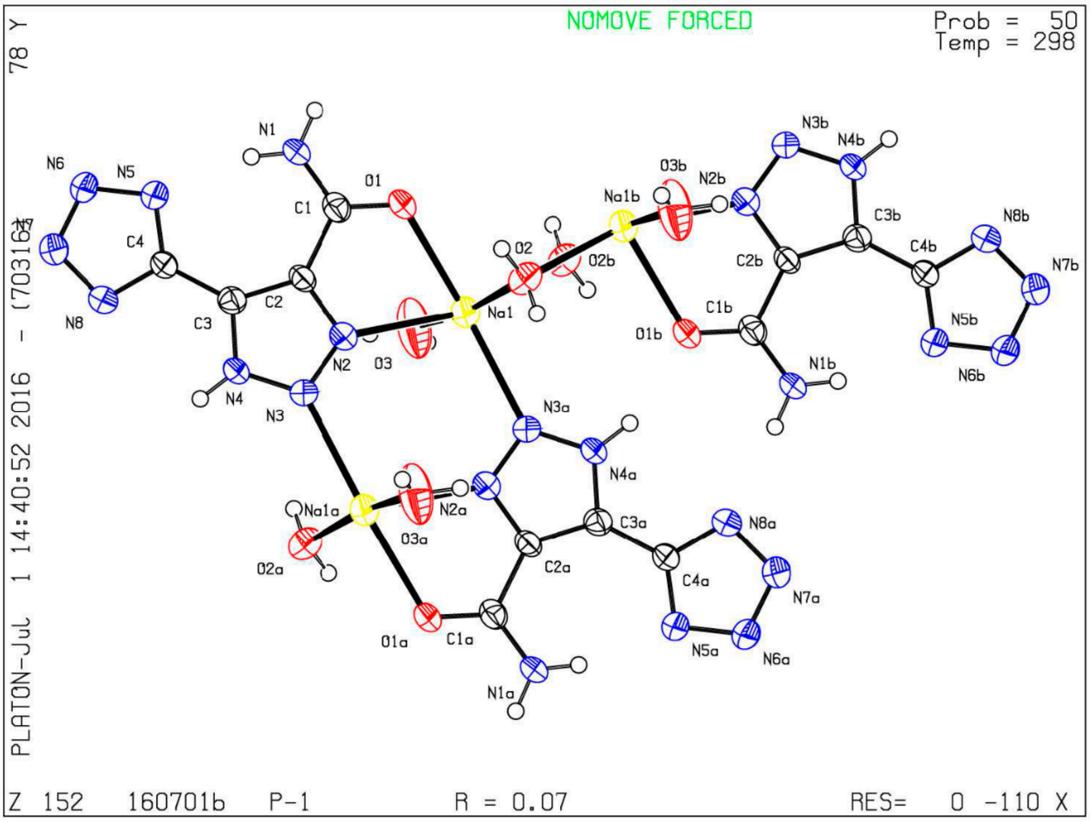

Figure S3. crystal structure of **4.**

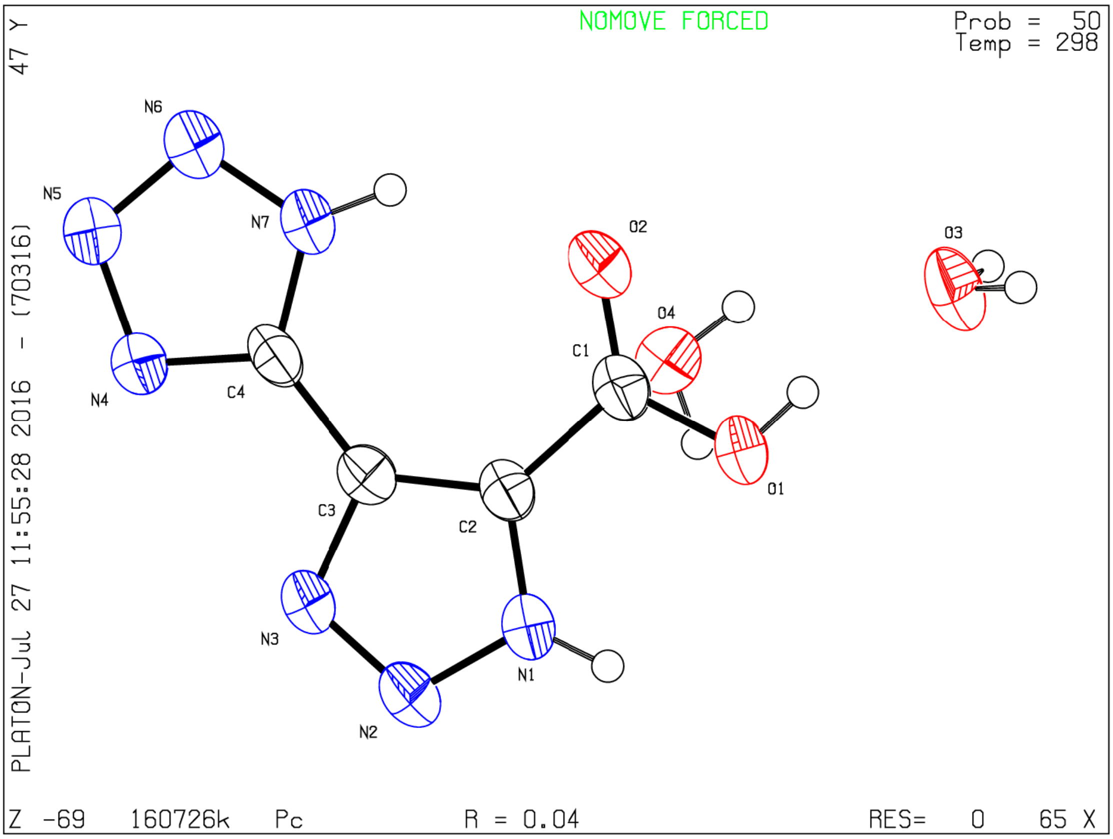

Figure S4. crystal structure of **6.**

Table S3. Crystal data and structure refinement for compounds 7 ~ 10.

	**7**	**8**	**9**	**10**
Empirical formula	$C_4H_7N_3O_6$	$C_6H_7N_3O_4$	$C_4H_9N_7O_3$	$C_4H_5N_5O_3$
MW (g mol^{-1})	193.13	185.15	203.18	171.13
Temperature (K)	298(2)	296(2)	298(2)	298(2)
Crystal System	Monoclinic	Triclinic	Monoclinic	Monoclinic
Space Group	P2(1)/c	P-1	P2(1)/c	C2/c
a (Å)	6.9034(5)	6.726(4)	4.5230(4)	6.7730(6)
b (Å)	17.1130(16)	9.197(5)	16.3569(13)	11.4681(9)
c (Å)	6.6112(4)	14.369(8)	11.4481(9)	9.1379(8)
α (°)	90	102.463(14)	90	90
β (°)	94.4480(10)	98.312(15)	99.002(2)	111.027(3)
γ (°)	90	110.762(12)	90	90
V (Å)^3	778.68(10)	787.5(7)	836.52(12)	662.51(10)
Z	4	4	4	4
ρ_{calcd} (g cm^{-3})	1.647	1.562	1.613	1.716
μ (mm^{-1})	0.156	0.133	0.137	0.148
F (000)	400	384	424	352
θ (°)	2.38 – 25.01	1.50 – 24.99	2.19 – 25.01	3.55 – 25.02
Reflections collected	3789	4689	4065	1629
Unique reflections	1363	2695	1478	583
R_{int}	0.0505	0.0466	0.0595	0.0174
Data/restraints/parameters	1363 / 0 / 119	2695 / 0 / 239	1478 / 0 / 127	583 / 0 / 57
GOF on F^2	1.110	1.011	1.093	1.094
R_1, wR_2 [$I>2\sigma(I)$]	R_1=0.0512, wR_2=0.1362	R_1=0.1056, wR_2=0.2821	R_1=0.0639, wR_2=0.1514	R_1=0.0344, wR_2=0.0909
R_1, wR_2 (all data)	R_1=0.0720, wR_2=0.1550	R_1=0.1230, wR_2=0.2988	R_1=0.1117, wR_2=0.1677	R_1=0.0400, wR_2=0.0950
CCDC	1879826	1879827	1879828	1879817

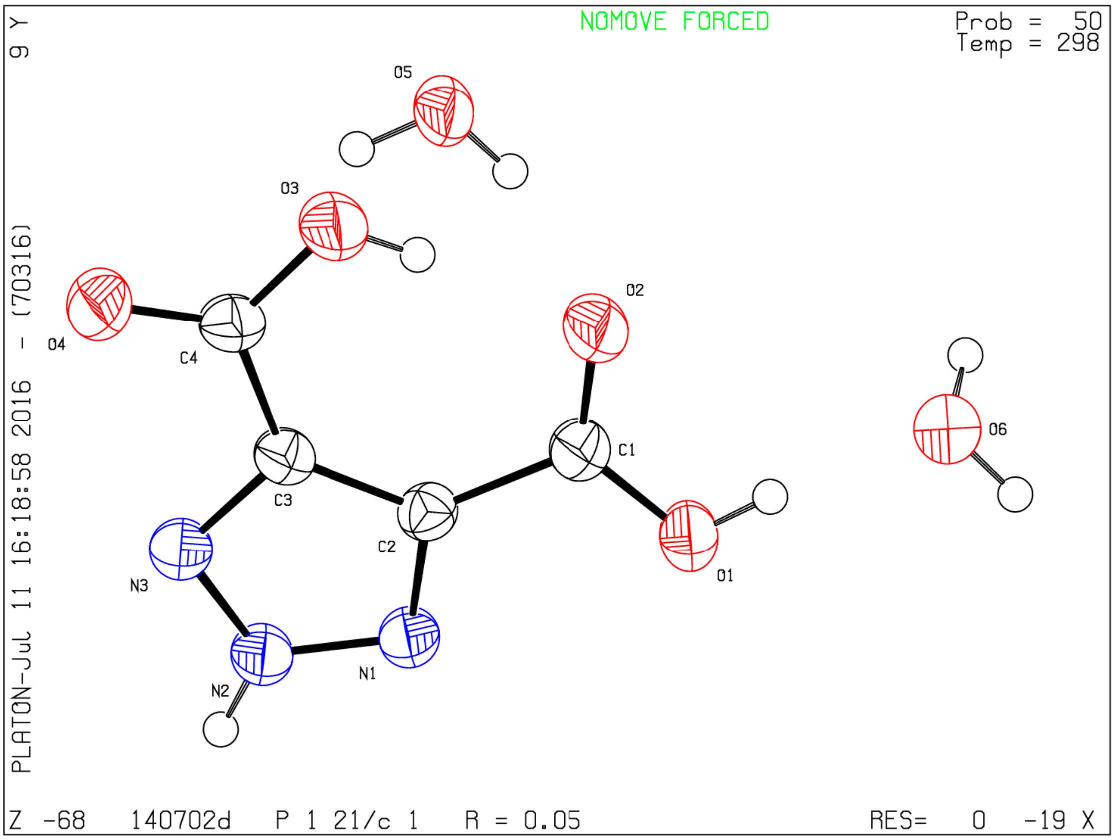

Figure S5. crystal structure of **7.**

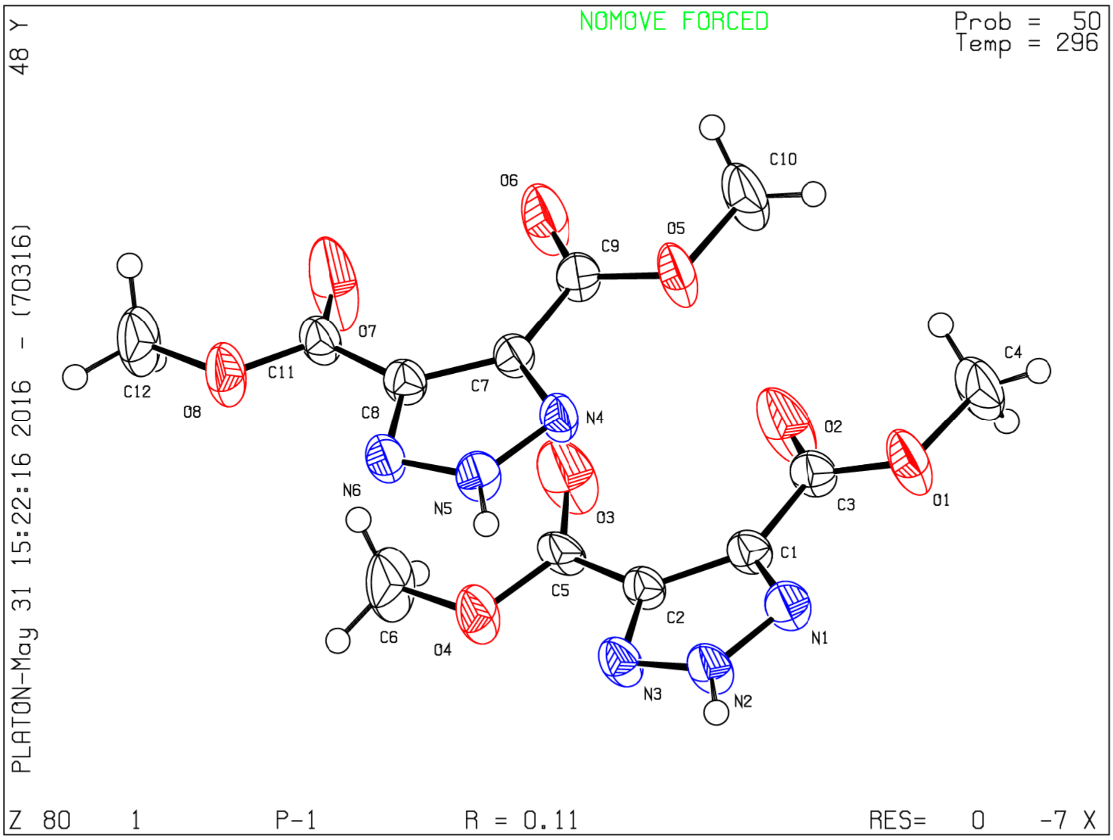

Figure S6. crystal structure of **8.**

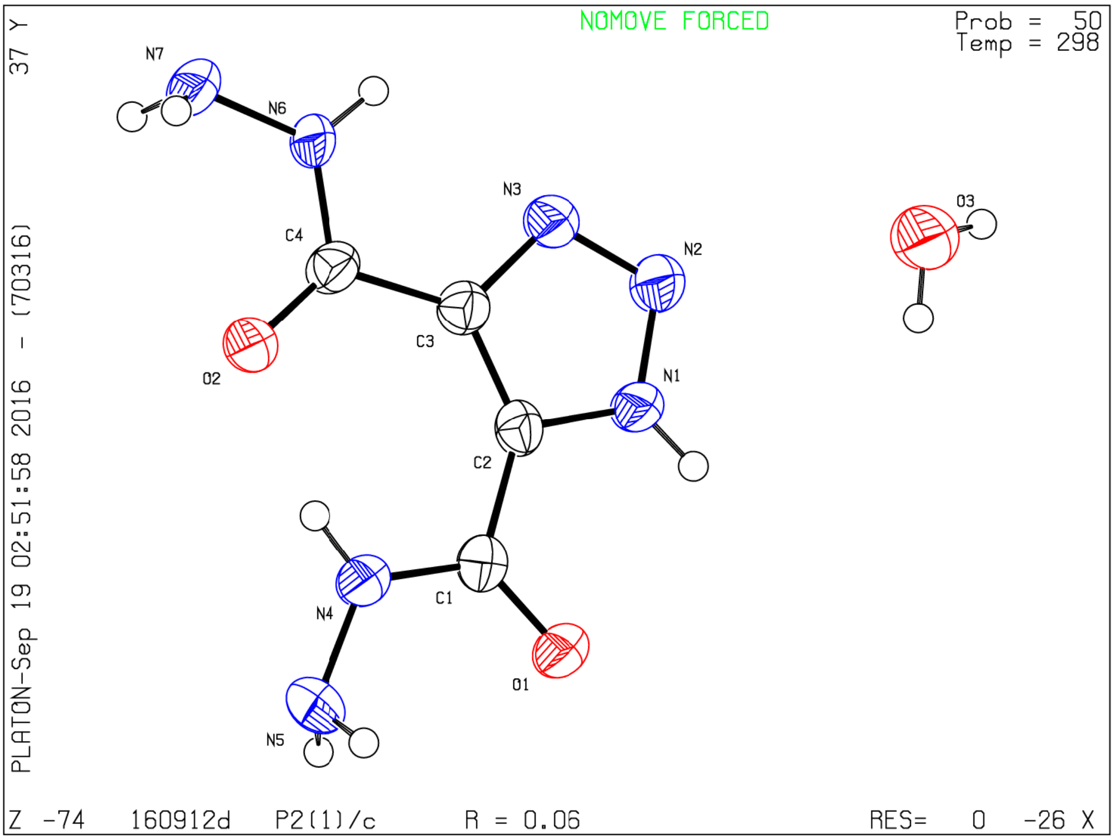

Figure S7. crystal structure of **9.**

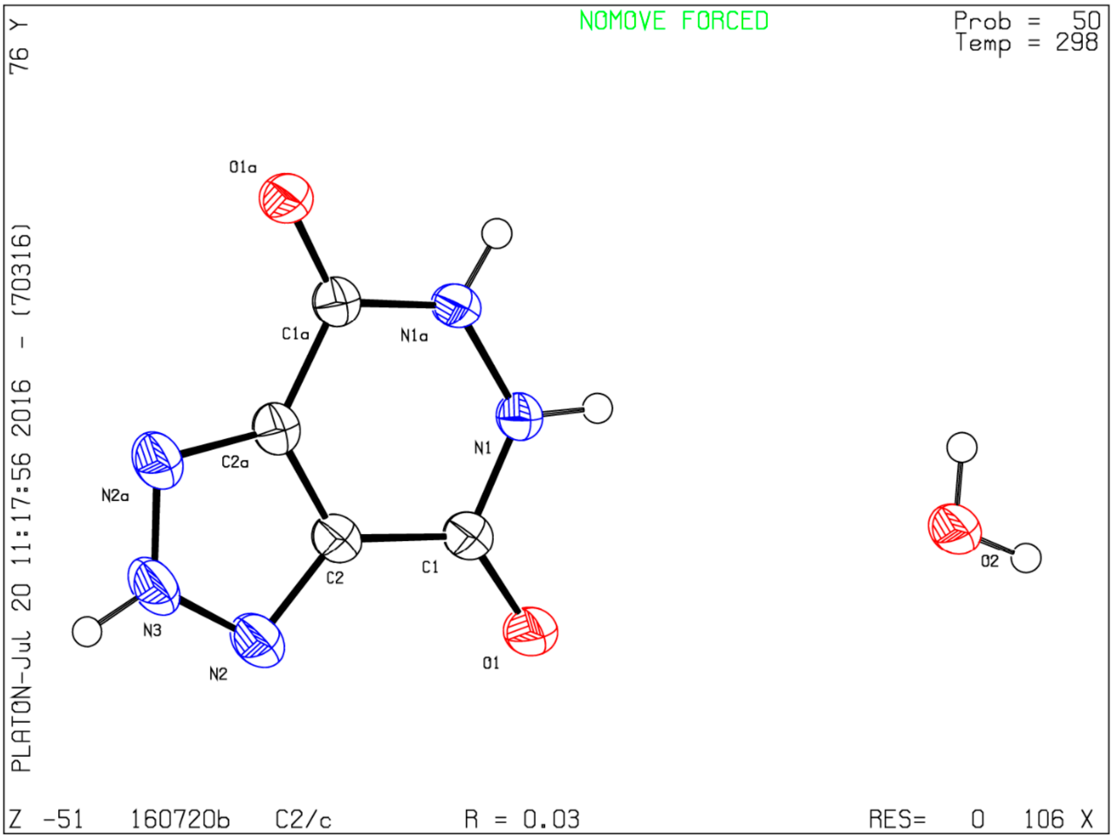

Figure S8. crystal structure of **10.**

Table S4. Crystal data and structure refinement for compounds 11 ~ 16.

	**11**	**12**	**13**
Empirical formula	$C_5H_3N_5$	$C_5H_7N_5O_2$	$C_5H_7N_3O_5$
MW (g mol^{-1})	133.12	169.16	189.14
Temperature (K)	298(2)	298(2)	293(2)
Crystal System	Orthorhombic	Monoclinic	Monoclinic
Space Group	Pca2(1)	P2(1)/n	C2/c
a (Å)	15.9113(13)	7.6416(7)	7.338(4)
b (Å)	7.4502(6)	6.2741(6)	13.429(7)
c (Å)	5.4677(4)	29.195(2)	16.375(8)
α (°)	90	90	90
β (°)	90	93.3990(10)	93.653(10)
γ (°)	90	90	90
V $(Å)^3$	648.15(9)	1397.3(2)	1610.3(14)
Z	4	8	8
ρ_{calcd} (g cm^{-3})	1.364	1.608	1.560
μ (mm^{-1})	0.097	0.129	0.141
F (000)	272	704	784
θ (°)	2.56 – 25.01	2.72 – 25.02	2.49 – 25.54
Reflections collected	3015	6754	4319
Unique reflections	1105	2468	1499
R_{int}	0.0447	0.1233	0.0390
Data/restraints/parameters	1105 / 1 / 92	2468 / 0 / 219	1499 / 5 / 131
GOF on F^2	1.005	1.079	0.938
R_1, wR_2 [$I>2\sigma(I)$]	R_1=0.0460, wR_2=0.1093	R_1=0.0774, wR_2=0.1634	R_1=0.0423, wR_2=0.1168
R_1, wR_2 (all data)	R_1=0.0841, wR_2=0.1259	R_1=0.1468, wR_2=0.1794	R_1=0.0529, wR_2=0.1283
CCDC	1879818	1879819	1879820

	**14**	**15**	**16**
Empirical formula	$C_7H_9N_3O_4$	$C_{20}H_{38}N_{28}O_9$	$C_5H_5N_5O_2$
MW (g mol^{-1})	199.17	814.78	167.14
Temperature (K)	298(2)	296(2)	298(2)
Crystal System	Monoclinic	Monoclinic	Triclinic
Space Group	P2(1)	P2/c	P-1
a (Å)	3.9100(3)	13.605(2)	3.8300(3)
b (Å)	7.4759(7)	3.8339(6)	8.1261(7)
c (Å)	15.5301(13)	32.858(5)	10.8879(9)
α (°)	90	90	94.1250(10)
β (°)	96.4300(10)	93.499(4)	99.488(2)
γ (°)	90	90	98.190(2)
V (Å)^3	451.10(7)	1710.7(5)	329.24(5)
Z	2	2	2
ρ_{calcd} (g cm^{-3})	1.466	1.582	1.686
μ (mm^{-1})	0.122	0.128	0.136
F (000)	208	852	172
θ (°)	2.64 – 25.00	1.242 – 24.998	2.54 – 25.02
Reflections collected	1465	10178	1698
Unique reflections	1465	3027	1152
R_{int}	0	0.0543	0.0293
Data/restraints/parameters	1465 / 1 / 132	3027 / 12 / 299	1152 / 0 / 110
GOF on F^2	1.239	0.973	1.211
R_1, wR_2 [$I>2\sigma(I)$]	R_1=0.0982, wR_2=0.2978	R_1=0.0499, wR_2=0.1216	R_1=0.0969, wR_2=0.3003
R_1, wR_2 (all data)	R_1=0.1119, wR_2=0.3083	R_1=0.0754, wR_2=0.1421	R_1=0.1170, wR_2=0.3103
CCDC	1879821	1879822	1879823

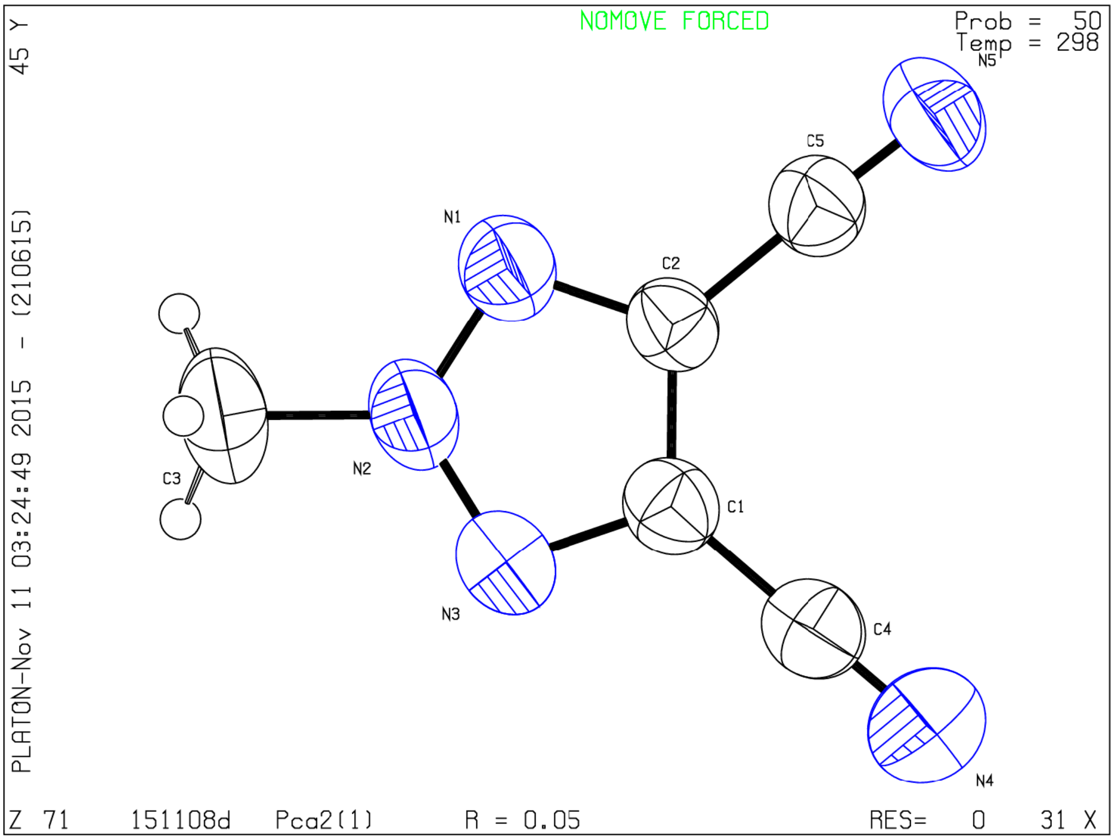

Figure S9. crystal structure of **11.**

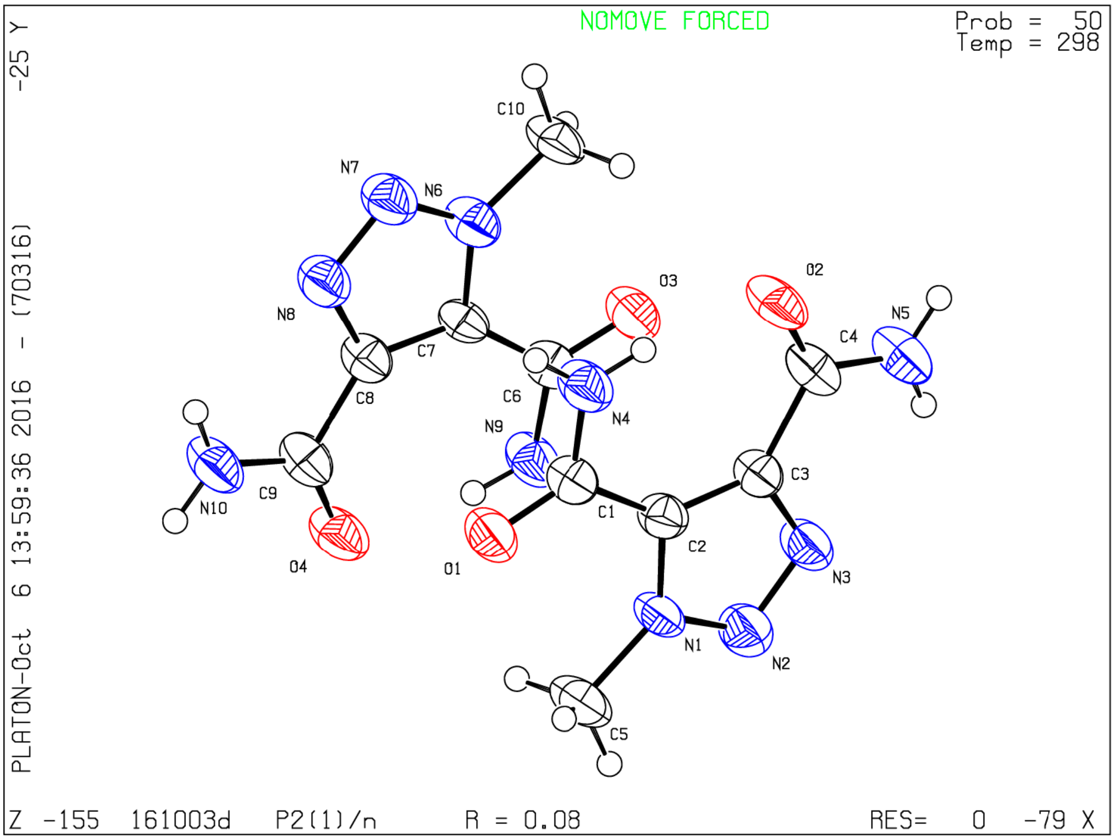

Figure S10. crystal structure of **12.**

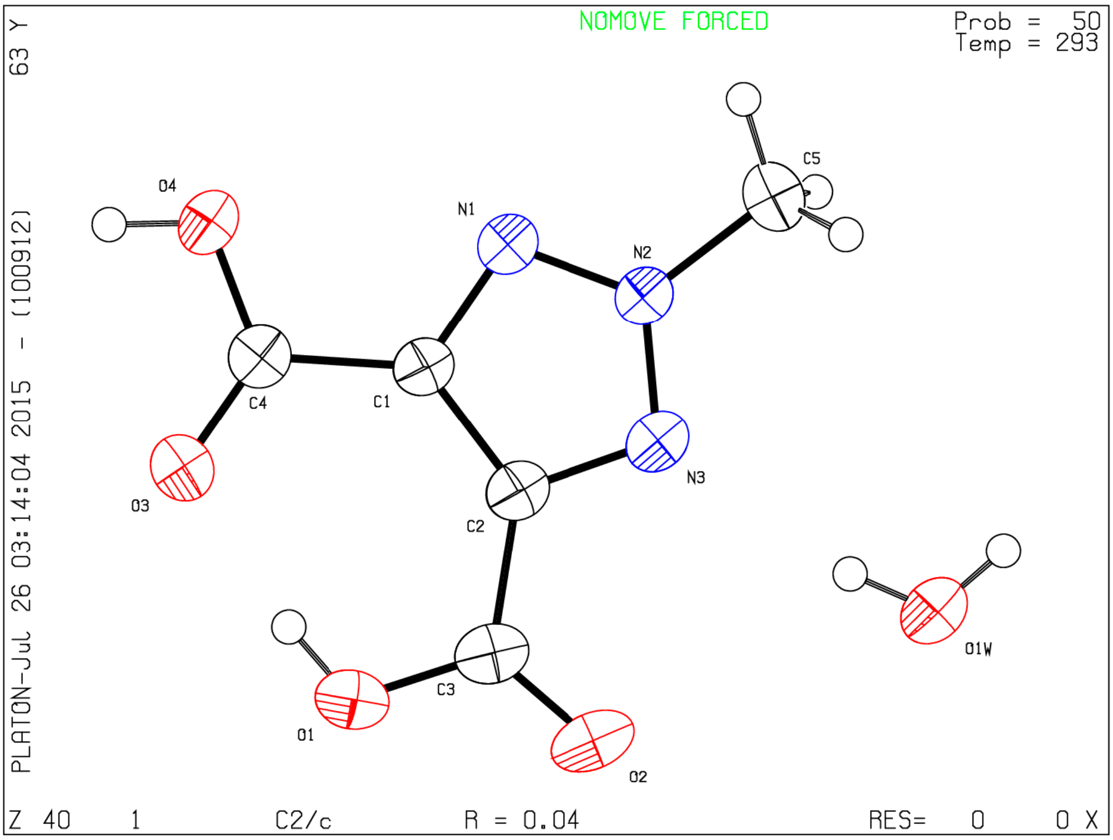

Figure S11. crystal structure of **13.**

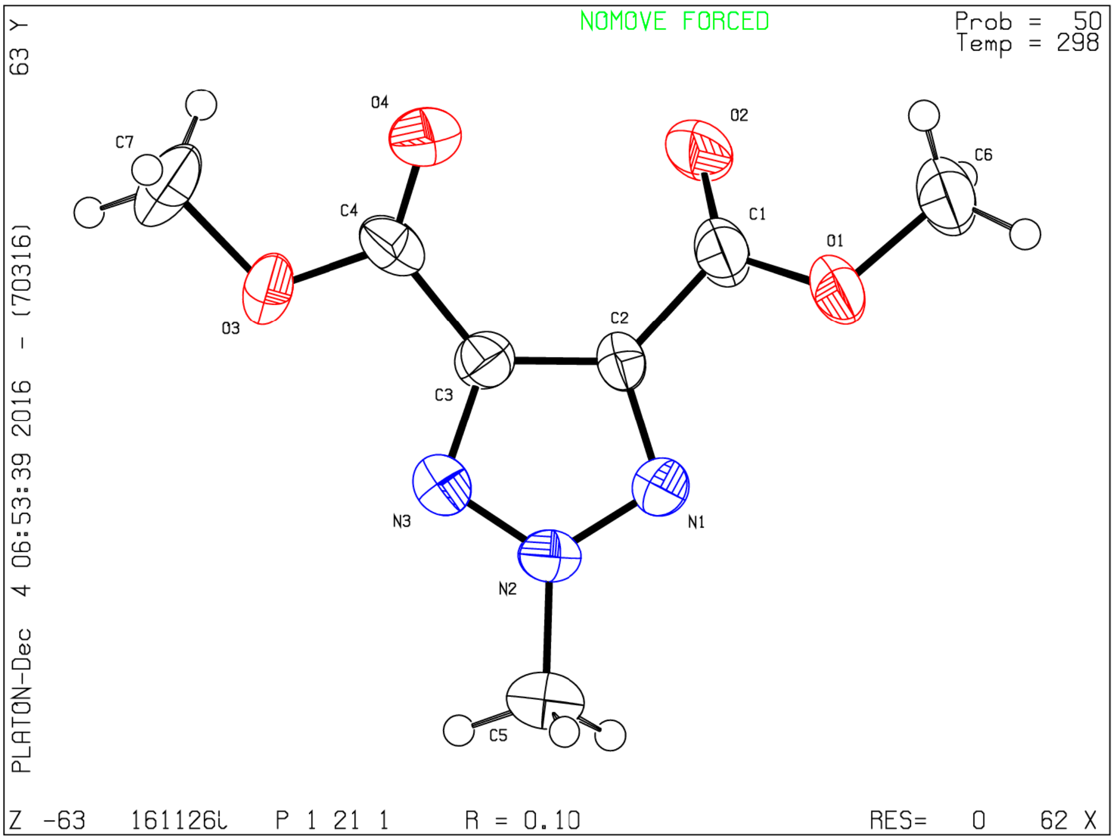

Figure S12. crystal structure of **14.**

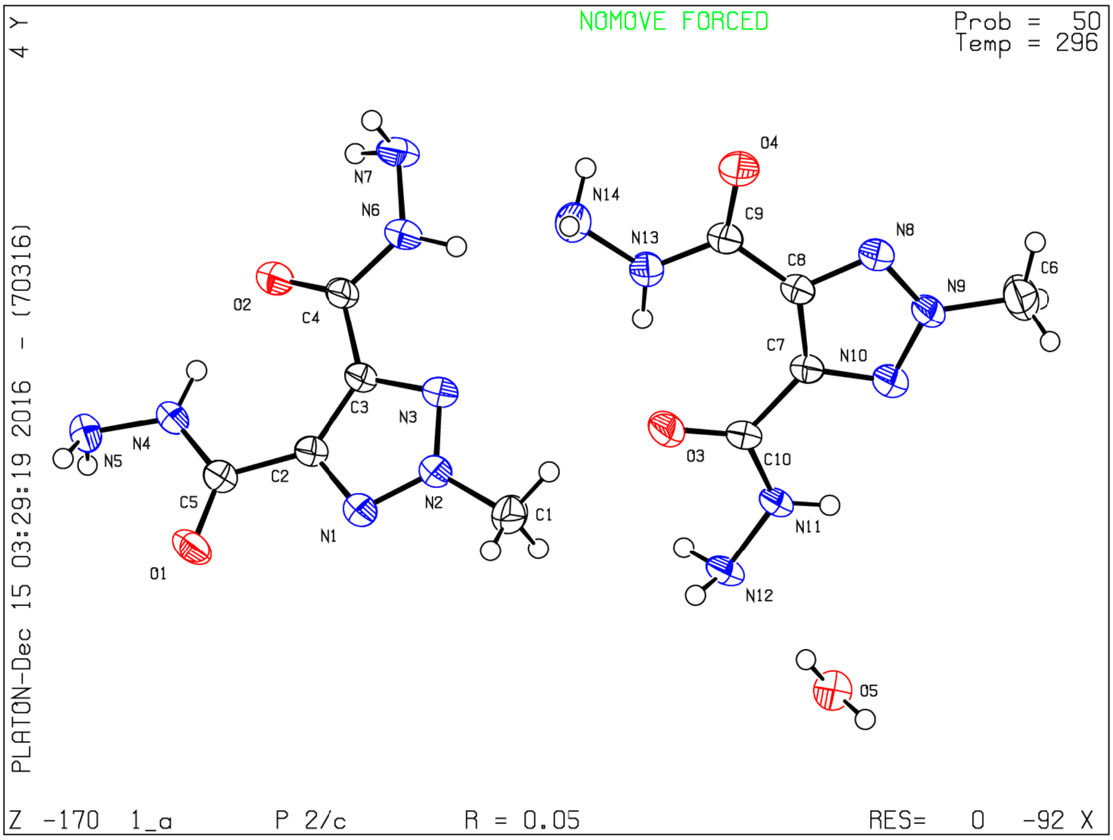

Figure S13. crystal structure of **15.**

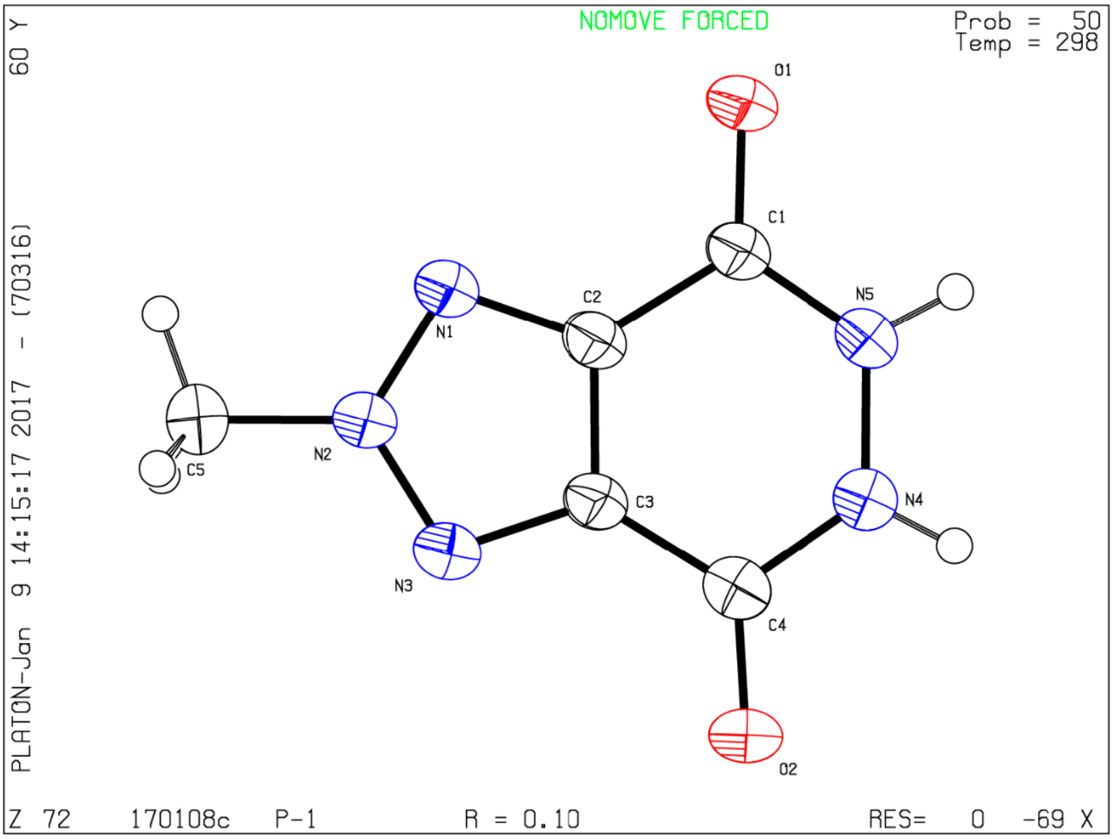

Figure S14. crystal structure of **16.**
